# A critical review of natural products driven correction of bile acid dysregulation: a therapeutic strategy for nonalcoholic fatty liver disease

**DOI:** 10.3389/fphar.2025.1640873

**Published:** 2025-11-21

**Authors:** Qing Peng, Liyuan Hao, Shenghao Li, Fei Yu, Na Li, Xiaoyu Hu

**Affiliations:** 1 Clinical Medical College, Chengdu University of Traditional Chinese Medicine, Chengdu, Sichuan, China; 2 Department of Infectious Diseases, Hospital of Chengdu University of Traditional Chinese Medicine, Chengdu, Sichuan, China; 3 Department of Integrated Traditional Chinese and Western Medicine Oncology, the Fourth Hospital of Hebei Medical University, Shijiazhuang, Hebei, China

**Keywords:** nonalcoholic fatty liver disease, bile acids, metabolism, farnesoid X receptor, natural products

## Abstract

Nonalcoholic fatty liver disease (NAFLD) represents a significant global health challenge. While two drugs (semaglutide, resmetirom) have recently been approved for nonalcoholic steatohepatitis (NASH), their clinical utility is constrained by gastrointestinal side effects, insufficient efficacy against fibrosis, and dose-related adverse events. Similarly, obeticholic acid (OCA), a farnesoid X receptor (FXR) agonist with antifibrotic potential, is associated with significant side effects, including severe pruritus. Dysregulation of bile acid (BA) metabolism is a central driver of NAFLD progression, characterized by imbalances in synthesis, impaired enterohepatic circulation, and aberrant nuclear receptor signaling. Certain hydrophobic BAs contribute to hepatocyte apoptosis, oxidative stress, and inflammation, thereby exacerbating liver injury. Targeting BA homeostasis is thus a promising therapeutic strategy, with natural products emerging as attractive candidates due to their multi-target actions and favorable safety profiles. This review summarizes 10 major classes of natural products, including traditional Chinese medicine (TCM) formulas, flavonoids, saccharides, saponins, alkaloids, curcuminoids, lignans, iridoid glycosides, sterols/terpenoids, and phenolic acids/other phenolics, that alleviate NAFLD by regulating BA metabolism. These agents modulate BA-sensing receptors, reshape the gut microbiota to optimize BA conversion, and regulate key BA transporters and enzymes. Compared with synthetic drugs, natural products offer broader efficacy, lower toxicity, and greater adaptability to the heterogeneity of NAFLD. However, significant limitations persist. Preclinical studies rely heavily on single-sex rodent models, while clinical evidence remains inconsistent. Crucially, mechanistic causality, such as the interplay between the gut microbiota and BAs, lacks rigorous validation through methods like fecal microbiota transplantation (FMT) or gene knockout studies. Furthermore, challenges in metabolite standardization and dose rationality hinder clinical translation. Future research must prioritize human-relevant models, large-scale randomized controlled trials (RCTs) with histological endpoints, and robust causal validation. By addressing these gaps, natural products targeting BA metabolism hold great promise to complement or replace existing therapies, offering safer and more effective personalized treatments for NAFLD.

## 1 Introduction

Nonalcoholic fatty liver disease (NAFLD) is an acquired metabolic stress liver injury characterized by excessive hepatocellular lipid accumulation, closely related to insulin resistance and genetic susceptibility (Chalasani et al., 2018). The global prevalence of NAFLD exceeds 35% (Younossi et al., 2023). NAFLD begins with hepatic steatosis and can progress to nonalcoholic steatohepatitis (NASH), fibrosis, cirrhosis, and hepatocellular carcinoma (HCC) (Tilg et al., 2021). Notably, cirrhosis prevalence in NASH patients reaches up to 20%, making it the second leading cause of liver transplantation (Noureddin et al., 2018). Despite this substantial burden, dietary modification combined with physical activity remains the gold standard for managing disease progression in patients with nonalcoholic fatty liver (NAFL) without inflammation or fibrosis, prior to the consideration of pharmacotherapies approved for more advanced disease stages (Houttu et al., 2021). These challenges highlight the urgent need to explore effective pharmacological preventive and therapeutic strategies.

To develop such strategies, a deeper understanding of pathogenesis is crucial. NAFLD is a metabolic disorder closely linked to obesity, diabetes, lipid metabolism abnormalities, hepatic bile acid (BA) dysregulation, and insulin resistance (Tilg et al., 2021; Neuschwander-Tetri, 2020). Reflecting its systemic metabolic nature, NAFLD was renamed metabolic dysfunction-associated steatotic liver disease (MASLD) in 2023, a shift that underscores the central role of metabolic dysregulation in its pathogenesis (Kanw et al., 2024). As NAFLD remains the predominant term in the cited literature, this review will adopt it hereafter. For years, the therapeutic landscape for NASH was characterized by a significant gap, dominated by numerous agents under investigation that often faced setbacks due to limited efficacy or safety concerns. For instance, Obeticholic acid (OCA), the farnesoid X receptor (FXR) agonist targeting BA signaling, showed efficacy in improving fibrosis, but its clinical use is hampered by 50% incidence of pruritus (Younossi et al., 2019). Next-generation FXR agonists, such as vonafexor and tropifexor, were developed to improve safety but still contend with dose-related adverse effects (Ratziu et al., 2023; Sanyal et al., 2023). Recently, the field has been transformed by the first regulatory approvals for NASH. However, even these groundbreaking agents are not without limitations. The GLP-1 receptor agonist semaglutide, while significantly improving hepatic inflammation and steatosis, exhibits a high incidence of gastrointestinal side effects and shows limited efficacy for fibrosis in the overall population (Sanyal et al., 2025). Similarly, thyroid hormone receptor-β (THR-β) agonist resmetirom, which demonstrates superior efficacy in achieving NASH resolution and fibrosis improvement, is associated with an increased risk of gastrointestinal side effects like diarrhea and nausea (Harrison et al., 2024). Thus, even these groundbreaking agents do not offer a universal solution, underscoring a continued need for therapies with broader efficacy and improved tolerability.

In this context, targeting BA homeostasis, one of the most critical metabolic dysfunctions driving NAFLD progression, remains a promising strategy, but one that demands a more nuanced and tolerable approach than that offered by current agents. Natural products, bioactive metabolites derived from plants, fungi, and marine organisms, have emerged as a compelling solution. Their multi-target effects and historical use in traditional Chinese medicine (TCM) to treat metabolic disorders make them uniquely suited to modulate BA homeostasis holistically. Specifically, natural products ameliorate BA dysregulation through coordinated regulation of BA synthetic enzymes, receptor-mediated signaling, and gut microbiota-dependent metabolic remodeling, thereby attenuating NAFLD progression. In this review, we synthesize recent advances in BA dysregulation mechanisms and evaluate natural products capable of modulating BA metabolism to ameliorate NAFLD.

## 2 Search strategy and study selection

A systematic and rigorous approach was employed to ensure a comprehensive and unbiased selection of relevant studies. Literature searches were conducted across multiple databases, including PubMed, Web of Science, Scopus, and ClinicalTrials.gov. The search strategy utilized a combination of keywords such as natural products, bile acid, nonalcoholic fatty liver disease, nonalcoholic steatohepatitis, and pharmacological activities, integrated with Boolean operators (AND, OR) to refine and optimize search results.

Studies were selected based on the following inclusion criteria: (Chalasani et al., 2018): investigation of natural products for the prevention or treatment of NAFLD/NASH, (Younossi et al., 2023), publication in peer-reviewed journals, (Tilg et al., 2021), presentation of original experimental (*in vitro*, *in vivo*) or clinical findings, and (Noureddin et al., 2018) availability in English. Exclusion criteria included non-English publications, review articles, meta-analyses, or letters without original data, studies unrelated to hepatic diseases or natural products, and research exclusively focused on synthetic metabolites. The selection process involved an initial screening of titles and abstracts to eliminate irrelevant studies, followed by a thorough full-text review to confirm eligibility based on the predefined inclusion and exclusion criteria.

## 3 NAFLD pathogenesis: mechanisms and therapeutic challenges

**FIGURE 1:**
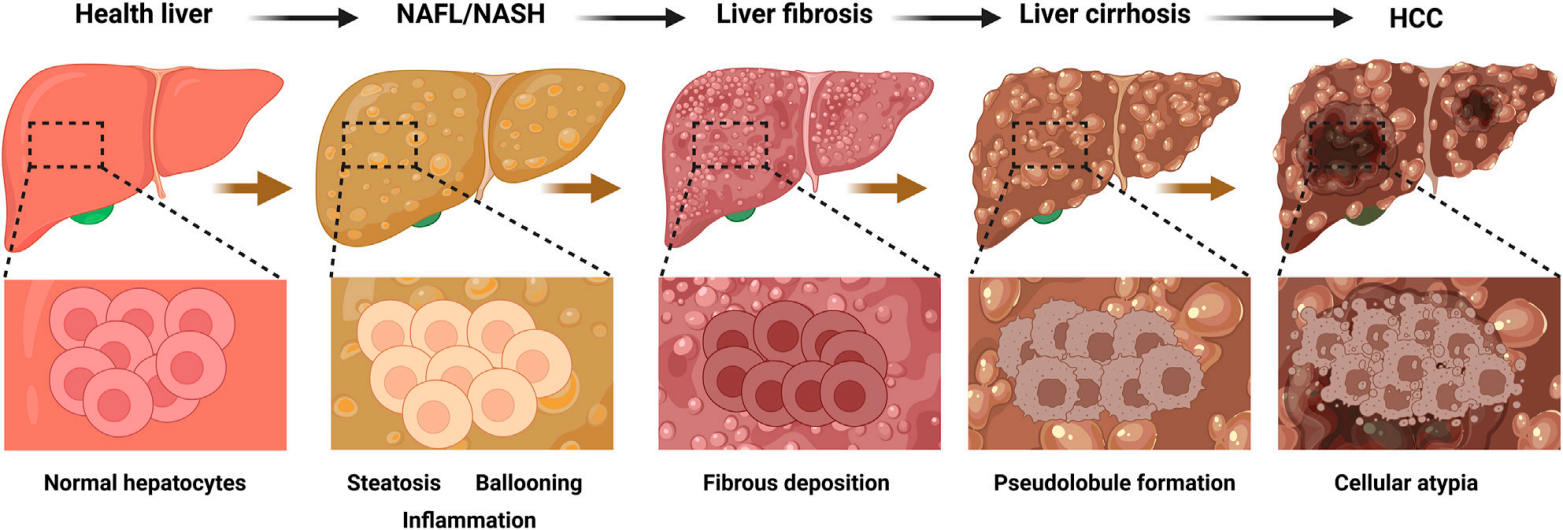
Progression of liver disease and corresponding histopathological changes. This figure illustrates the sequential development of liver diseases from a healthy state to HCC), with the top row depicting the macroscopic appearance of the liver at each stage and the bottom row showing the corresponding histopathological features at higher magnification. The progression begins with a healthy liver, characterized by normal hepatic architecture and uniform-sized hepatocytes. This advances to NAFL/NASH, which is characterized by lipid accumulation within hepatocytes (steatosis), swelling of hepatocytes (ballooning degeneration), and infiltration of inflammatory cells. Subsequently, liver fibrosis develops, marked by the excessive deposition of extracellular matrix proteins that form fibrous septa and disrupt the normal liver structure. In the next stage, liver cirrhosis, this advanced fibrosis leads to the formation of regenerative nodules surrounded by fibrous tissue, creating an abnormal, nodular architecture (pseudolobules). Ultimately, the process culminates in HCC, where malignant transformation is indicated by significant cellular atypia, including a loss of normal cellular organization, enlarged and irregular nuclei, and uncontrolled proliferation. HCC, hepatocellular carcinoma; NAFL, nonalcoholic fatty liver disease; NASH: nonalcoholic steatohepatitis. The figure was created in https://BioRender.com.

NAFLD, the most common chronic liver disease globally and a leading cause of liver-related complications and mortality (Teng et al., 2023), is defined by hepatic steatosis ≥5% (confirmed by imaging or histology) after excluding secondary causes such as alcohol, drugs, or viral hepatitis (Chalasani et al., 2012). While elevated liver enzymes (ALT or AST) may be present, they are not diagnostic prerequisites (Chalasani et al., 2012). NAFLD manifests as two distinct subtypes: NAFL, characterized by isolated steatosis (≥5%) without significant inflammation or hepatocellular damage (Brunt et al., 2021), and NASH, a progressive form marked by lobular inflammation, hepatocyte ballooning, and fibrosis, which can progress to severe fibrosis, cirrhosis, or HCC (Brunt et al., 2021) (Figure 1).

Although extensive research has been conducted on the pathogenesis of NAFLD, its complex and multifactorial mechanisms are not yet fully elucidated, which has hindered the development of effective treatments. Early studies proposed the two-hit hypothesis, wherein insulin resistance and hepatic steatosis caused by excess fatty acids represent the first hit, while subsequent hepatocyte damage, driven by oxidative stress and lipid peroxidation, triggers inflammation, fibrosis, and other pathological changes, collectively termed the second hit (Basaranoglu et al., 2013). The later multiple-hit theory expanded this model by incorporating oxidative stress, endoplasmic reticulum stress, and lipotoxicity (Buzzetti et al., 2016). It is now established that NAFLD pathogenesis is closely linked to a network of metabolic disturbances, including obesity, insulin resistance, gut dysbiosis, inflammation, and disruptions in BA and lipid metabolism (Murag et al., 2021).

Several FXR agonists, including OCA, vonafexor, and tropifexor, have shown efficacy in improving NASH pathology, yet each is also burdened by distinct adverse effects. Nonetheless, the therapeutic efficacy of these agents underscores the critical role of FXR in BA metabolism, positioning it as an important target for NAFLD therapy. FXR, a nuclear receptor, is predominantly expressed in hepatocytes and enterocytes (Nijmeijer et al., 2014). As an endogenous BA sensor identified in 1999 (Makishima et al., 1999), FXR maintains BA homeostasis by suppressing cholesterol 7α-hydroxylase (CYP7A1), the rate-limiting enzyme in BA synthesis, thereby preventing hepatotoxic BA accumulation (Myant and Mitropoulos, 1977). However, NAFLD disrupts this delicate equilibrium by altered BA composition, impaired enterohepatic circulation, and aberrant signaling, all of which drive progression to cirrhosis and HCC (Lai et al., 2023; Xue et al., 2021). Thus, regulation of BA signaling pathways remains a promising therapeutic target for NAFLD.

## 4 BAs metabolism

### 4.1 Synthesis

**FIGURE 2:**
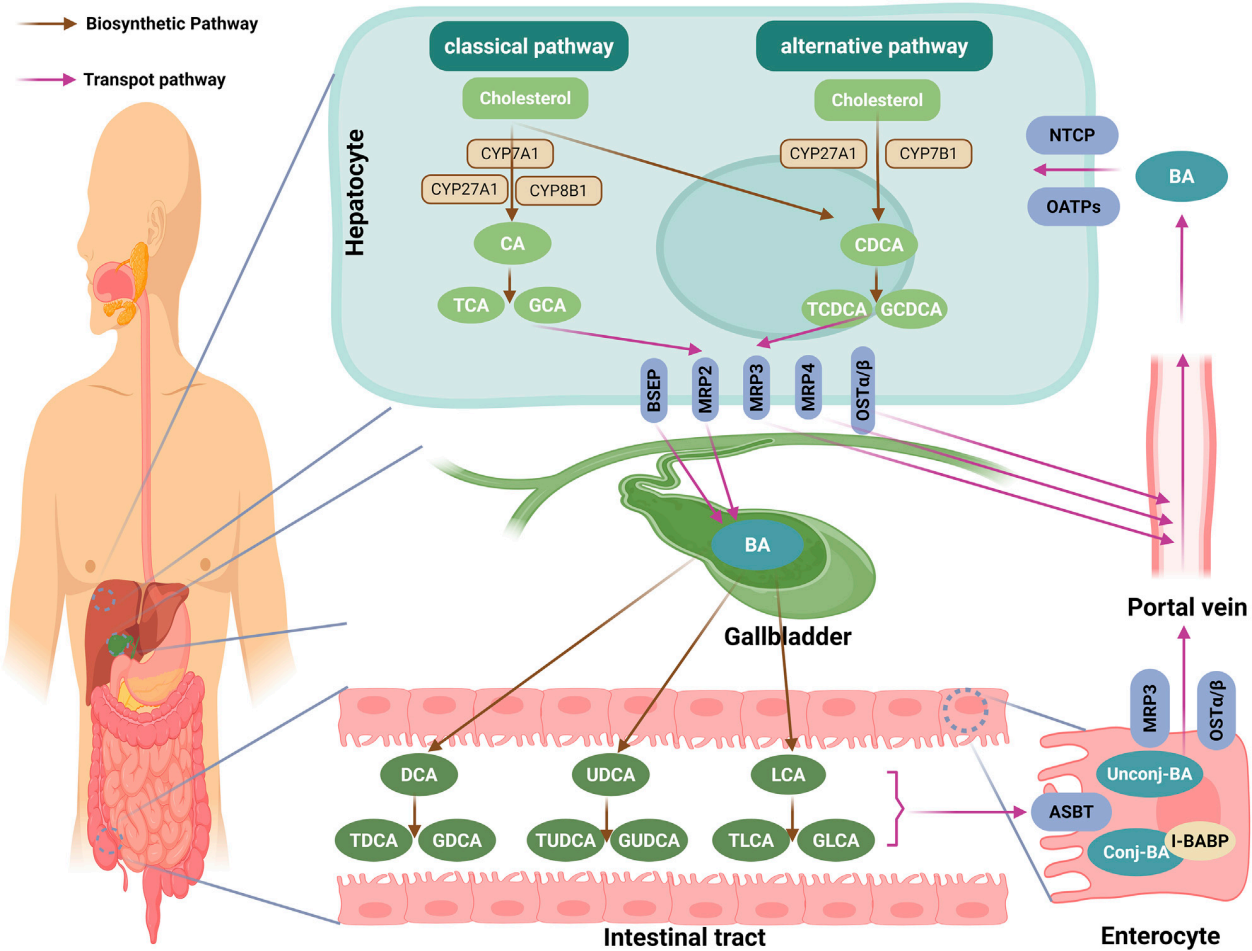
Biosynthesis and enterohepatic circulation of BAs. This diagram encapsulates the intricate pathway of BA metabolism, highlighting key steps from synthesis to circulation. BAs are synthesized from cholesterol in hepatocytes via two pathways: the classical (neutral) pathway, initiated by the rate-limiting enzyme CYP7A1, and the alternative (acidic) pathway, initiated by CYP27A1. Both pathways converge to produce the primary BAs, CA and CDCA, which are then conjugated with taurine (form TCA and TCDCA) or glycine (form GCA and GCDCA). These conjugated BAs are actively secreted into bile canaliculi primarily by the BSEP, with multidrug MRP2 facilitating the excretion of organic anions and sulfated BAs. In cholestatic conditions, hepatocytes utilize basolateral transporters like OSTα/β, MRP3, and MRP4 to redirect BAs into the systemic circulation, circumventing biliary obstruction. Following storage in the gallbladder, they are released postprandially into the duodenum to aid lipid absorption. In the intestine, the vast majority (∼95%) of BAs are reabsorbed in the terminal ileum via the ASBT. Within enterocytes, BAs bind to I-BABP and are exported into the portal blood through basolateral transporters like OSTα/β and MRP3. The small fraction of BAs that reaches the colon undergoes transformation by the gut microbiota, including deconjugation and 7α-dehydroxylation, converting primary BAs into secondary forms such as DCA, LCA or UDCA. These secondary BAs can then be reconjugated with glycine or taurine by the host liver or certain gut bacteria, forming conjugated secondary BAs such as GDCA, TDCA, GLCA, TLCA, GUDCA, and TUDCA. Finally, hepatocytes recapture the BAs from the portal circulation via NTCP and OATPs, completing the enterohepatic circulation. Through this continuous cycle, the composition of the human BA pool stabilizes to approximately 40% CA, 40% CDCA, and 20% DCA. BA, bile acid; CYP7A1: cholesterol 7α-hydroxylase; CA, cholic acid; CDCA, chenodeoxycholic acid, TCA, taurocholic acid; TCDCA, taurochenodeoxycholic acid; GCA, glycocholic acid; GCDCA, glycochenodeoxycholic acid; BSEP, bile salt export pump; MRP2, multidrug resistance-associated protein 2; OSTα/β, organic solute transporter α/β; MRP3, multidrug resistance-associated protein 3; MRP4, multidrug resistance-associated protein 4; ASBT, apical sodium-dependent BA transporter; I-BABP, ileal BA-binding protein; DCA, deoxycholic acid; LCA, lithocholic acid; UDCA, ursodeoxycholic acid; GDCA, glycodeoxycholic acid; TDCA, taurodeoxycholic acid; GLCA, glycolithocholic acid; TLCA, taurolithocholic acid; GUDCA, glycoursodeoxycholic acid; TUDCA, tauroursodeoxycholic acid; NTCP, basolateral sodium taurocholate co-transporting polypeptide; OATPs, organic anion-transporting polypeptides; The figure was created in https://BioRender.com.

BAs are synthesized from free cholesterol in hepatocytes via two pathways: the classical (neutral) pathway (responsible for ∼90% of BA synthesis) and the alternative (acidic) pathway (contributing ≤10%) (Jackson et al., 2022). However, this ratio is not static. Recent evidence indicates it is dynamically regulated by physiological cues such as circadian rhythm and nutritional status (Yang et al., 2024a; Mohanty et al., 2024). In the classical pathway, CYP7A1, exclusively expressed in hepatocytes, acts as the rate-limiting enzyme, converting cholesterol to 7α-hydroxycholesterol, which is then transformed into 7α-hydroxy-4-cholesten-3-one (C4) by a hydroxysteroid dehydrogenase (Chiang and Ferrell, 2020). C4 is converted into cholic acid (CA) via hydroxylation mediated by cytochrome P450 family 8 subfamily B member 1 (CYP8B1), or into chenodeoxycholic acid (CDCA) in the absence of CYP8B1 (Chiang and Ferrell, 2020). The alternative pathway, initiated by the mitochondrial enzyme cholesterol 27α-hydroxylase (CYP27A1), converts cholesterol into 27-hydroxycholesterol and 3β-hydroxy-5-cholestenoic acid, which are further hydroxylated by cytochrome P450 family 7 subfamily B member 1 (CYP7B1), and subsequently transported to the liver for conversion into CA and CDCA (Chiang and Ferrell, 2020). Both pathways converge at the production of CA and CDCA, which are conjugated with glycine to form glycocholic acid (GCA) and glycochenodeoxycholic acid (GCDCA), or with taurine to form taurocholic acid (TCA) and taurochenodeoxycholic acid (TCDCA), for storage in the gallbladder (Režen et al., 2022) (Figure 2).

Postprandial gallbladder contraction releases conjugated BAs into the small intestine to facilitate lipid digestion; approximately 95% of these are reabsorbed in the distal ileum via the enterohepatic circulation (Li and Chiang, 2014; Wu et al., 2021). The remaining 5% reach the colon, where gut microbiota (*Bacteroides*, *Clostridium*) first hydrolyze conjugated BAs via bile salt hydrolases (BSH) and then 7α-dehydroxylate the resulting free CA/CDCA via the enzyme 7α-dehydroxylase, generating the secondary BAs (deoxycholic acid (DCA) from CA, and lithocholic acid (LCA) or ursodeoxycholic acid (UDCA) from CDCA) (Jia et al., 2018) (Figure 2).

These secondary BAs can then be reconjugated with glycine or taurine by the host liver or certain gut bacteria, forming conjugated secondary BAs such as glycodeoxycholic acid (GDCA), taurodeoxycholic acid (TDCA), glycolithocholic acid (GLCA), taurolithocholic acid (TLCA), glycoursodeoxycholic acid (GUDCA), and tauroursodeoxycholic acid (TUDCA), which are then reabsorbed or excreted. Beyond deconjugation and dehydroxylation, the gut microbiota can perform additional transformations, including epimerization and oxidation of the hydroxyl groups, further diversifying the BA pool (He et al., 2024a; Har et al., 2018). These newly formed secondary BAs, along with any unabsorbed primary BAs, are then taken up by the liver and rejoin the enterohepatic circulation (Li and Chiang, 2014). After multiple enterohepatic cycles, the composition of the human BA pool stabilizes to approximately 40% CA, 40% CDCA, and 20% DCA (Li and Chiang, 2014; Guo et al., 2022) (Figure 2).

### 4.2 Transport

Primary BAs, once conjugated with taurine or glycine, are actively secreted into bile canaliculi via the bile salt export pump (BSEP), a hepatocyte canalicular membrane transporter that mediates ATP-dependent transport of conjugated BAs (Boyer, 2013). Concurrently, multidrug resistance-associated protein (MRP) 2 facilitates the biliary excretion of organic anions and sulfated BAs into bile ducts (König et al., 1999). Under cholestatic conditions, hepatocytes employ basolateral transporters, specifically the organic solute transporter (OST) α/β, MRP3, and MRP4, to export BAs into the systemic circulation, thereby bypassing biliary obstruction (Chiang and Ferrell, 2020). After being concentrated in the gallbladder, BAs are released postprandially into the duodenum to facilitate lipid absorption (Li and Chiang, 2014). Approximately 95% of these BAs are reabsorbed in the terminal ileum through the apical sodium-dependent BA transporter (ASBT) (Oelkers et al., 1997). Intracellularly, BAs bind ileal BA-binding protein (I-BABP) and are effluxed into the portal blood via basolateral OSTα/β and MRP3 (Rao et al., 2008; Marin et al., 2015). Finally, hepatocytes recapture portal BAs through basolateral sodium taurocholate co-transporting polypeptide (NTCP) and organic anion-transporting polypeptides (OATPs) (Meier and Stieger, 2002), completing the enterohepatic circulation (Figure 2).

### 4.3 Regulation and dysregulation in NAFLD

**FIGURE 3:**
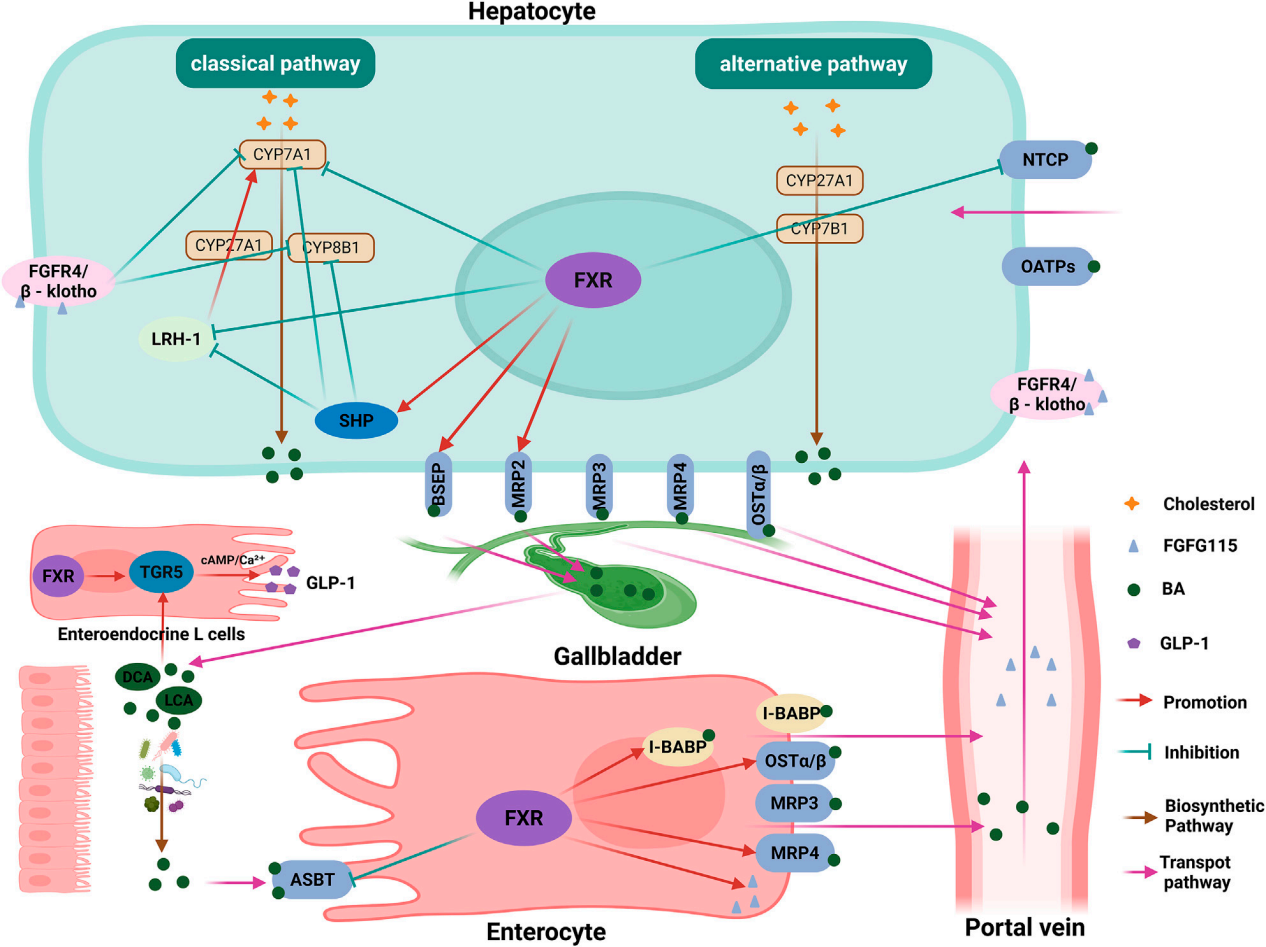
FXR-mediated regulation of BA homeostasis and metabolism. This diagram illustrates the central regulatory network governing bile acid (BA) homeostasis, orchestrated primarily by the nuclear receptor FXR. In hepatocytes, elevated BA levels activate FXR, which induces the expression of SHP. SHP subsequently suppresses the transcription of the rate-limiting enzyme CYP7A1 and the cholic acid-specific enzyme CYP8B1, thereby inhibiting BA synthesis. This suppression is mediated through the disruption of HNF4α and LRH-1 transcriptional activity. Concurrently, intestinal FXR activation stimulates the secretion of FGF15, which, upon binding to the hepatic FGFR4/β-Klotho complex, initiates a signaling cascade that further represses CYP7A1 and CYP8B1 transcription. Beyond synthesis, FXR coordinates BA transport by upregulating canalicular exporters (BSEP, MRP2) and downregulating the basolateral uptake transporter NTCP in the liver, while in the intestine, it induces efflux transporters (OSTα/β, MRP4) and I-BABP, and suppresses the apical uptake transporter ASBT. These actions collectively promote BA excretion and limit reabsorption. Separately, secondary BAs (DCA, LCA) activate the G protein-coupled receptor TGR5 on enteroendocrine L cells, triggering cAMP/Ca^2+^-dependent secretion of GLP-1. This enhances insulin sensitivity and stimulates systemic energy metabolism. FXR, farnesoid X receptor; BA, bile acid; SHP, small heterodimer partner; CYP7A1, cholesterol 7α-hydroxylase; CYP8B1, sterol 12α-hydroxylase; HNF4α, hepatocyte nuclear factor 4 alpha; LRH-1, liver receptor homolog-1; FGF15, fibroblast growth factor 15; FGFR4, fibroblast growth factor receptor 4; BSEP, bile salt export pump; MRP2, multidrug resistance-associated protein 2; OSTα/β, organic solute transporter α/β; MRP4, multidrug resistance-associated protein 4; I-BABP, ileal bile acid binding protein; DCA, deoxycholic acid; LCA, lithocholic acid; TGR5, takeda G protein-coupled receptor 5; GLP-1, glucagon-like peptide-1. The figure was created in https://BioRender.com.

Under normal conditions, the BA pool is maintained through coordinated actions of nuclear receptors and transporters. FXR, a ligand-activated nuclear receptor predominantly expressed in hepatocytes and enterocytes, serves as the master regulator of BA homeostasis by controlling synthesis, transport, and detoxification pathways (Forman et al., 1995; Matsubara et al., 2013). Specifically, elevated BA levels activate hepatic FXR, which induces small heterodimer partner (SHP) to repress the transcription of CYP7A1 (the rate-limiting enzyme in BA synthesis) through two mechanisms: disrupting hepatocyte nuclear factor 4α (HNF4α) binding to the BA response element-I (BARE-I) and amplifying suppression via direct interaction with liver receptor homolog-1 (LRH-1) (Lu et al., 2000; Li and Chiang, 2020). Concurrently, FXR/SHP signaling downregulates CYP8B1 (the enzyme specific for cholic acid enzyme) transcription in hepatocytes (Chiang and Ferrell, 2020). Furthermore, intestinal FXR activation stimulates fibroblast growth factor 15 (FGF15) secretion; upon binding to hepatic fibroblast growth factor receptor 4 (FGFR4) in complex with its co-receptor β-Klotho, FGF15 initiates a signaling cascade that predominantly suppresses CYP7A1 while synergistically inhibiting CYP8B1 transcription, thereby reducing BA synthesis (Kong et al., 2012; Inagaki et al., 2005). FXR also orchestrates BA transport by upregulating canalicular exporters (BSEP, MRP2) and downregulating the basolateral uptake transporter NTCP in hepatocytes, while inducing I-BABP and basolateral efflux transporters (OSTα/β, MRP4) and suppressing ASBT in the intestine, actions that collectively promote BA excretion and limiting reabsorption (Chiang and Ferrell, 2020; Ananthanarayanan et al., 2001; Denson et al., 2001; Kast et al., 2002; Cai and Boyer, 2006). Beyond BA homeostasis, secondary BAs (DCA, LCA) activate the membrane receptor takeda G protein-coupled receptor 5 (TGR5), triggering cAMP/Ca^2+^ dependent secretion of glucagon-like peptide-1 (GLP-1) from enteroendocrine L cells to enhance insulin sensitivity, stimulate adipose tissue browning, and improve systemic energy metabolism (Chiang and Ferrell, 2020). In addition to its metabolic roles, TGR5 is now recognized to have broader physiological functions, including regulating gallbladder filling and bile flow through its expression on gallbladder epithelial and smooth muscle cells (Holter et al., 2020) (Figure 3).

However, this tightly regulated system is profoundly disrupted in NAFLD. The circulating BA profile is significantly altered, with these changes correlating with NAFLD severity (Puri et al., 2018). Compared with healthy subjects, NAFLD patients exhibit elevated total BA levels, which progressively increase from NAFL to NASH (Jiao et al., 2018; Nimer et al., 2021). A higher ratio of secondary to primary BAs, along with increased levels of conjugated BAs, is also correlated with fibrosis risk (Puri et al., 2018). Notably, specific conjugated BAs such as GCA, TCA, GCDCA, and TCDCA show stepwise increases from healthy individuals to NAFL, and further to NASH (Puri et al., 2018). This plasma BA elevation is particularly pronounced in NASH patients with insulin resistance and remarkably, TCA and GCA levels can effectively distinguish NASH independent of obesity or insulin resistance (Grzych et al., 2021). Similarly, elevated serum ratios of GCA/TCA, GDCA/TDCA, and GCDCA/TCDCA are observed in early-stage NAFLD/NASH (Chen et al., 2022). Crucially, these changes associate with histological severity, including steatosis, lobular and portal inflammation, and hepatocyte ballooning, thereby directly linking BA dysregulation to core aspects of liver pathophysiology (Hylemon et al., 2021).

The underlying mechanisms of this dysregulation are complex and multifaceted. Mechanistically, during liver fibrosis progression in NASH mice, hepatocytes redirect BAs from biliary secretion to systemic circulation by regulating the expression of BA synthesis enzymes (CYP7A1) and transporter (BSEP, NTCP and OSTβ), leading to increased systemic BA concentrations (Suga et al., 2019). Furthermore, inflammation-induced downregulation of BA transporters further impairs enterohepatic circulation, exacerbating hepatic and serum BA accumulation (Hylemon et al., 2021). Additionally, an increased ratio of FXR-antagonistic DCA to FXR-agonistic CDCA suppresses the FXR-FGFR4 signaling pathway manifesting as CYP7A1 upregulation, NTCP reduction and decreased fibroblast growth factor 19 (FGF19) levels (Jiao et al., 2018). This creates a self-perpetuating cycle of BA accumulation and signaling failure, ultimately driving NAFLD progression. Therefore, targeting BA metabolism represents a promising therapeutic strategy.

## 5 Potential natural products for target BA metabolism in NAFLD

**FIGURE 4:**
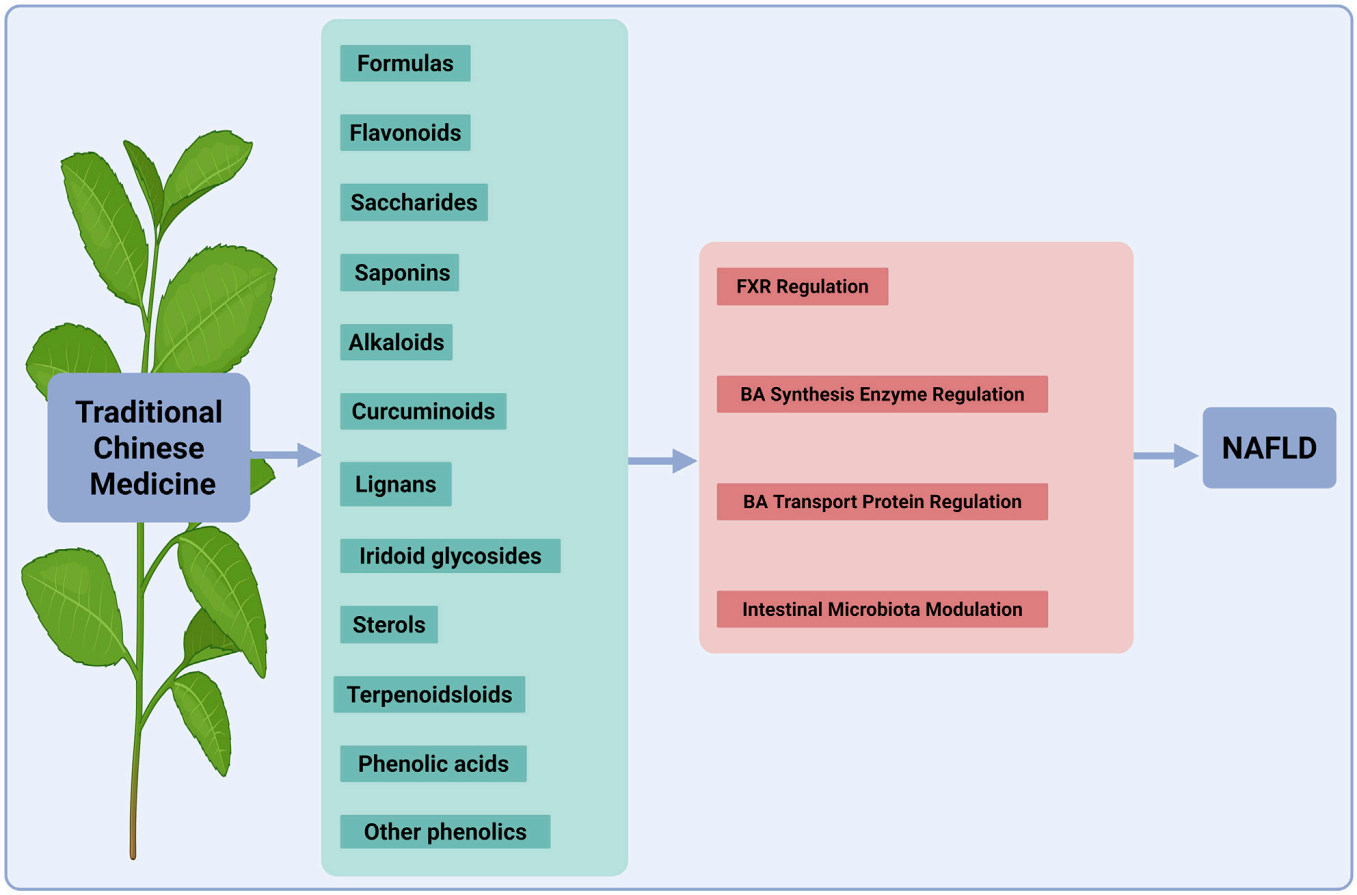
Mechanisms of TCM components in regulating BA metabolism for NAFLD therapy. This diagram illustrates the mechanisms by which various TCM components treat NAFLD by regulating BA metabolism. The active ingredients shown, such as formulas, flavonoids, saccharides, saponins, alkaloids, curcuminoids, lignans, iridoid glycosides, sterols/terpenoids and phenolic acids/other phenolics, target key pathways. These include the nuclear receptor FXR, the BA synthesis enzyme, BA transporters, and the intestinal microbiota. Collectively, these actions restore BA homeostasis to alleviate NAFLD. TCM, traditional Chinese medicine; NAFLD, nonalcoholic fatty liver disease; BA, bile acid; FXR, farnesoid X receptor. The figure was created in https://BioRender.com.

NAFLD represents a burgeoning global health crisis with limited effective pharmacotherapies. Patients with NAFLD exhibit disorders of BAs homeostasis (Lai et al., 2023), characterized by altered synthesis, impaired transport and dysregulated receptor signaling. These disturbances potentiate the progression from simple steatosis to more severe conditions, such as steatosis to cirrhosis and HCC (Xue et al., 2021). In this section, we summarized recent advances in the use of natural products and their active metabolites to treat NAFLD by modulating BAs metabolism (Figure 4).

### 5.1 TCM formulas

Some commonly used clinical TCM formulas, historically applied to treat liver, gallbladder, or other diseases, have recently demonstrated efficacy against NAFLD by regulating BA metabolism (Table 1). The botanical composition and taxonomic validation for all TCM formulas are summarized in Supplementary Table S1.

**TABLE 1:** TCM formulas for target BA metabolism in NAFLD.

Number	Agents	Dose	Experimental subject	Molecular targets	References
1	Jiangzhi granule	0.12, 0.24 g/kg/d	male C57BL/6 J	intestinal FXR↑	Cao et al. (2022)
2	LG decoction	715 mg/kg/d	male C57BL/6	ileum FGF15/FXR↑; liver CYP7A1↓	Chen et al. (2024)
3	LG oral solution	2.5, 5.0, 10.0 g/kg/d	male mice	ileum FGF15/FXR↑; liver FXR↑, CYP7A1↓	Wang et al. (2024a)
4	LG decoction	12 g Fuling, 9 g Guizhi, 6 g Baizhu, and 6 g Gancao/d or 1/2 dose	human	/	Dai et al. (2022)
5	Zhuyu pill	0.75, 1.5, 3 g/kg	male C57BL/6	FXR/FGF15↓; CYP7A1↑	Xu et al. (2023)
6	PCP extracts	2, 4, 8 g/kg	male C57BL/6 J	BSH-producing bacteria↓; ileal FXR/FGF15↓; FXR/BSEP/CYP27A1↑	Li et al. (2022a)
7	PCBG	2.5, 5, 10 μM	L02 cells	liver FXR/ BSEP↑; liver CYP7A1↓	Li et al. (2022b)
8	PCB	1, 5, 25 μM	L02 cells	liver FXR/ BSEP↑; liver CYP7A1↓	Li et al. (2022b)
9	Huazhi-Rougan formula	3, 6 g/kg/d	male C57BL/6 J	ileal ASBT and OSTβ↓	Li et al. (2022c)
10	Zexie-Baizhu Decoction	1500 mg/kg/d	male C57BL/6 J	remodeling the gut microbiota	Shi et al. (2025)
11	Xiaohua Funing decoction	18 g/kg/d	male Wistar	remodeling the gut microbiota	Li and Zhao. (2025)
12	Gan-Jiang-Ling-Zhu Decoction	1.8, 3.6, 7.2 g/kg/d	male C57BL/6 J	remodeling the gut microbiota	Ma et al. (2025)
13	Qiang-Gan formula	400 mg/kg	male C57BL/6	liver TGR5, BSEP and MRP2↑; BA-producing genera↑	Li et al. (2020a)
14	Qianggan capsule	10 capsules/d	human	/	Li et al. (2010)
15	Qinlian Hongqu Decoction	0.51,1.02, 2.04 g/kg/d	male C57BL/6J	intestinal FXR/TGR5/GLP-1↑;hepatic CYP7A1↑	Zhang et al. (2024a)
16	Huaganjian decoction	0.62,1.24, 2.48 mg/kg/d	male SD	ileal FXR ↓, FGF15 ↑; liver FXR/SHP2 ↑, CYP7A1 ↓	Do et al. (2025)

TCM, traditional Chinese medicine; BA, bile acid; NAFLD, nonalcoholic fatty liver disease; FXR, farnesoid X receptor; LG, Ling-Gui-Zhu-Gan; FGF15, fibroblast growth factor 15; CYP7A1, cholesterol 7α-hydroxylase; PCP, *Penthorum chinense* Pursh; BSEP, bile salt export pump; PCBG, pinostrobin-7-O-β-D-glucoside; PCB, metabolite pinocembrin; ASBT, apical sodium-dependent bile salt transporter; OSTβ, organic solute transporter β; TGR5, G-protein coupled bile acid receptor 1; GLP-1, glucagon-like peptide-1; SHP2, small heterodimer partner 2; SD, Sprague-Dawley.

A prominent therapeutic strategy involves the activation of intestinal FXR/FGF15 signaling to suppress hepatic BA synthesis. For instance, the Jiangzhi granule ameliorated NASH in mice by activating intestinal FXR to increase secondary BAs and strengthen gut barrier integrity (Cao et al., 2022). Similarly, Ling-Gui-Zhu-Gan (LG) decoction mitigated BA dysregulation in NAFLD mice through coordinated ileal FXR/FGF15 activation and hepatic CYP7A1 suppression (Chen et al., 2024), a finding supported by work on LG oral solution (Wang et al., 2024a). This approach was further supported by a trial where low-dose LG decoction with lifestyle therapy improved insulin resistance in mildly overweight NAFLD patients (Dai et al., 2022). However, these studies were largely confined to mouse models, lacked clinical validation, and did not investigate the specific molecular targets or active metabolites.

In contrast, a seemingly paradoxical strategy involves the suppression of intestinal FXR/FGF15 signaling, thereby disinhibiting hepatic CYP7A1 to accelerate BA synthesis and cholesterol clearance. For example, the Zhuyu pill paradoxically suppressed ileal FXR/FGF15 signaling, thereby upregulating hepatic CYP7A1 to accelerate BA synthesis and cholesterol clearance (Xu et al., 2023). This strategy aligned with the action of Gansukeli, derived from *Penthorum chinense* Pursh (PCP) extracts, which is clinically used for viral hepatitis (Li et al., 2022a). PCP extracts ameliorated NAFLD by reshaping the gut microbiota through the reduction of BSH-producing bacteria, which decreased BA deconjugation and increased fecal taurine-conjugated BAs, and by inhibiting ileal FXR/FGF15 while activating hepatic FXR/BSEP/CYP27A1 (Li et al., 2022a). However, a critical limitation of this study was the failure to determine the direct action of core metabolites, such as pinostrobin-7-O-β-D-glucoside (PCBG), on BSH-producing bacteria and FXR. This gap was particularly noteworthy given that a previous study reported the active metabolite PCBG and its metabolite pinocembrin (PCB) alleviated hepatic lipid accumulation in free fatty acids induced L02 cells by activating hepatic FXR/BSEP expression and inhibiting CYP7A1 expression (Li et al., 2022b). This discrepancy highlighted a recurring challenge across these studies, the failure to establish a direct and consistent link between the key active metabolites and their ultimate effects on the FXR pathways.

Beyond direct FXR modulation, many TCM formulas targeted other key nodes in the BA enterohepatic circulation. For example, Huazhi-Rougan formula inhibited ileal ASBT/OSTβ, reducing BA reabsorption and enhancing fecal excretion of toxic secondary BAs (Li et al., 2022c). Similarly, both Zexie-Baizhu Decoction (Shi et al., 2025) and Xiaohua Funing decoction (Li and Zhao, 2025) ameliorated hepatic lipid accumulation by restoring BA homeostasis, a process achieved through remodeling the gut microbiota (such as decreasing the Firmicutes/Bacteroidetes ratio and increasing Akkermansia) and modulating key BA pools across the gut-blood-liver axis. In addition, Gan-Jiang-Ling-Zhu Decoction improved lean MASLD by modulating the gut microbiota, a change characterized by increased abundance of phyla such as Verrucomicrobia and Bacteroidetes and decreased abundance of the pathogenic genus *Streptococcus*, which in turn promoted the fecal excretion of secondary BAs and simultaneously inhibited the reabsorption of toxic secondary BAs from the serum (Ma et al., 2025). Qiang-Gan formula enriched BA-producing genera like Clostridia and *Bacteroides*, stimulating fecal production of secondary BAs, and upregulated hepatic TGR5 and BA transporters (BSEP/MRP2) (Li et al., 2020a). In a multicenter randomized controlled trial, the Qianggan capsule reduced hepatic TG accumulation and serum ALT levels and improved the liver-to-spleen CT ratio, with no serious adverse events reported (Li et al., 2010).

Notably, some TCM formulas exhibit complex, multi-target actions that defy simple categorization. For instance, Qinlian Hongqu Decoction alleviated NAFLD by activating the intestinal FXR/TGR5/GLP-1 signaling pathways, yet paradoxically also increased the expression of hepatic CYP7A1 (Zhang et al., 2024a). Similarly, Huaganjian decoction restored gut-liver BA homeostasis by inhibiting ileal FXR, upregulating ileal FGF15, increasing hepatic FXR/SHP2, and decreasing hepatic CYP7A1 (Do et al., 2025). However, these studies were often restricted to male mice and failed to identify the specific active metabolites responsible for the observed effects.

Although these TCM formulas demonstrated clear efficacy in ameliorating NAFLD, the interpretation of their underlying mechanisms frequently presented seemingly contradictory phenomena, such as both the activation and inhibition of FXR. This inconsistency primarily stemmed from the inherent complexity of the TCM formulas and the limitations of the experimental designs. On one hand, the multi-metabolite, multi-target nature of these formulas meant that their various chemical metabolites could exert synergistic or antagonistic effects on the same pathway, leading to complex regulatory outcomes in the overall effect. On the other hand, most studies failed to identify the core active metabolites. Consequently, their mechanistic explorations were largely confined to correlational analyses of the gut microbiota, BAs, and pathway protein expression, and lacked causal confirmation experiments such as gene knockouts or receptor antagonists. This made it difficult to distinguish whether the observed effects were due to the holistic action of the formula or the direct action of specific metabolites. Furthermore, variations in animal models, including strain differences and modeling methods, also contributed to divergent pathway regulation. Therefore, the mechanistic contradictions observed were essentially a reflection of the transitional challenges that arise when applying relatively simplified research paradigms to dissect a highly complex system. Future research needs to delve into the level of individual active metabolites and incorporate more rigorous causal experiments to reveal a unified and definitive mechanistic logic.

### 5.2 Flavonoids

Flavonoids, widely distributed in plants, demonstrate multi-targeted therapeutic advantages in treating metabolic diseases, attributed to their unique structural features including polyphenolic hydroxyl groups and glycosylation modifications (Table 2).

**TABLE 2:** Flavonoids for target BA metabolism in NAFLD.

Number	Agents	Dose	Experimental subject	Molecular targets	References
1	Quercetin	25, 50, 100 mg/kg/d	Male SD	FXR ↑	Wang et al. (2024b)
2	Kaempferol	25, 50, 100 mg/kg/d	Male SD	FXR ↑	Wang et al. (2024b)
3	Quercetin	500 mg/d	Human	—	Li et al. (2024a)
4	Kaempferol	0.5 mL/100 g	Male C57BL/6J	CYP27A1, NTCP ↑	Lu et al. (2022)
5	Isorhamnetin	5 mg/kg	Male C57BL/6N	Liver FXR, BSEP ↑; OATP-1B3↓	La et al. (2024)
6	Isoquercetin	700 mg/kg/d	Male C57BL/6	CYP27A1, CYP7B1↑; intestinal FXR↓; liver BSEP, MRP2↑; ileal ASBT, I-BABP↓; harmful bacteria↓	Zhang et al. (2023)
7	Hyperoside	0.6,1.5 mg/kg/d	Male Wistar	Liver FXR ↑	Wang et al. (2021a)
8	Nobiletin	10, 50,100 mg/kg/d	Male C57BL/6J	CYP7A1, CYP27A1 ↑	Xu et al. (2024)
9	Tectorigenin	25, 50 mg/kg/d	Male C57BL/6N	Hepatic and intestinal FXR, CYP7A1 ↑; *Bacteroides*↑	Duan et al. (2022)
10	EGCG	—		Regulating intestinal flora	Ushiroda et al. (2019)
11	Epigallocatechin gallate	300 mg/d	Human	—	Yang et al. (2024b)
12	Flavan-3-ols	—	Male C57BL6/J	SHP↓; liver CYP7A1, CYP8B1↑	Vauzour et al. (2018)
13	Silibinin	100, 200 mg/kg/d	Male C57BL6/J	Regulating intestinal flora	Li et al. (2020b)
14	Silibinin	20 mg/kg/d	Male C57BL/6	Regulating intestinal flora	Wang et al. (2023)
15	Baicalein	100, 200 mg/kg/d	Male C57BL/6N	Regulating intestinal flora	Li et al. (2022d)
16	Silymarin	700 mg/d	Human	—	Wah Kheong et al. (2017)

BA, bile acid; NAFLD, nonalcoholic fatty liver disease; SD, Sprague-Dawley; FXR, farnesoid X receptor; CYP27A1, cholesterol 27α-hydroxylase; NTCP, sodium taurocholate co-transporting polypeptide; BSEP, bile salt export pump; OATP-1B3, organic anion transporting polypeptide 1B3; CYP7B1, cytochrome P450 family 7 subfamily B member 1; BSEP, bile salt export pump; MRP2, multidrug resistance-associated protein 2; ASBT, apical sodium-dependent bile salt transporter; I-BABP, ileal BA, binding protein; CYP7A1, cholesterol 7α-hydroxylase; EGCG, epigallocatechin-3-gallate; SHP, small heterodimer partner; CYP8B1, cytochrome P450 family 8 subfamily B polypeptide 1.

Quercetin, a prototypical flavonoid, was found to ameliorate NAFLD by activating FXR to regulate lipid metabolism, restore liver function, suppress inflammation, and mitigate oxidative stress (Wang et al., 2024b). However, the high-fat diet (HFD)/lipopolysaccharide rat model is a poor analog for human NAFLD, as it induces acute, exogenous inflammation rather than the chronic, metabolic condition and omits the critical gut-liver axis. A 12-week randomized, double-blind, placebo-controlled trial demonstrated that daily supplementation with 500 mg of quercetin moderately reduced intrahepatic lipid content in patients with NAFLD, accompanied by slight decreases in body weight and BMI, and the intervention showed good safety (Li et al., 2024a). Nevertheless, the trial is limited by its small sample size and a lack of long-term follow-up to assess the durability of effects and potential for delayed adverse events. In addition, while sharing FXR-dependent lipid modulation with quercetin (Wang et al., 2024b), kaempferol enhanced BA transport via upregulation of CYP27A1 and NTCP to adjust BA metabolism, suggesting potential efficacy against NASH progression (Lu et al., 2022). However, relying solely on male mice, while methodologically convenient, constitutes a flawed design that inappropriately generalizes male pathology to a diverse patient population. Building on quercetin’s framework, its metabolite isorhamnetin alleviated cholestasis and decelerated NAFLD progression by upregulating FXR-mediated BSEP to promote canalicular BA excretion, while OATP-1B3 suppression reduced sinusoidal reuptake, collectively shifting BA flux toward intestinal elimination to alleviate hepatic BA overload (La et al., 2024). Furthermore, the glycosylated derivative isoquercetin restored enterohepatic BA homeostasis not merely by mimicking quercetin but through selective pathway activation: it upregulated CYP27A1 and CYP7B1 without affecting CYP7A1/CYP8B1, inhibited intestinal FXR (not hepatic FXR), enhanced hepatic efflux transporters (BSEP/MRP2) and inhibited ileal reabsorption proteins (ASBT/I-BABP), while concurrently suppressing harmful gut bacteria (Zhang et al., 2023). The tissue specific FXR modulation, namely, activation by quercetin/isorhamnetin in the liver and inhibition by isoquercitrin in the gut, highlights a fragmented mechanistic landscape where the structural determinants of this selectivity are poorly defined, which in turn is a major barrier to the rational design of targeted NAFLD therapeutics.

Notably, hyperoside (quercetin-3-O-galactoside) diverged from its parent metabolite by preferentially increasing conjugated BA levels and reducing unconjugated BA toxicity, thereby amplifying hepatic FXR efficacy in rat (Wang et al., 2021a). Beyond quercetin derivatives, nobiletin, a polymethoxyflavone from citrus nobiletin, upregulated the expression of CYP7A1 and CYP27A1 and promoted a balanced intestinal microbiota, and reduced lipid levels, alleviating liver fat accumulation and improving NAFLD (Xu et al., 2024). Strikingly, tectorigenin, a methoxylated isoflavone with three hydroxyl groups, upregulated both hepatic and intestinal FXR and CYP7A1 expression, increased the relative abundance of specific *Bacteroides* species, thereby ameliorating serum BA accumulation and alleviating lipid-related symptoms associated with NAFLD (Duan et al., 2022). The translational relevance of the tectorigenin-driven microbiota-BA-flavonoid axis remains unproven, as the observed enrichment of *Bacteroides* in rodent models has not been validated in humans, whose gut microbiota differs significantly in composition.

Epigallocatechin-3-gallate (EGCG) has been shown to alleviate NAFLD by modulating the gut microbiome. In a preclinical study, EGCG alleviated NAFLD by enhancing taurine deconjugation and enriching beneficial taxa (Akkermansia) and inhibiting harmful taxa (Desulfovibrionaceae) (Ushiroda et al., 2019). However, this study only used male mice, and did not detect BA related molecules, nor did it verify the causal effect of the microbiota through fecal microbiota transplantation (FMT). A subsequent clinical trial then sought to translate these findings, demonstrating that daily oral administration of 300 mg of epigallocatechin gallate for 24 weeks significantly reduced hepatic fat content in patients with NAFLD (Yang et al., 2024b). Nevertheless, the study was limited by a small sample size and the absence of a placebo control, which meant that the generalizability and reliability of these findings required validation in larger-scale trials. Whereas flavan-3-ols alleviated NAFLD not by direct FXR agonism but through inhibiting SHP and upregulating CYP7A1 and CYP8B1 in the liver, while suppressing SHP and TGR5 in the intestine (Vauzour et al., 2018). However, the failure to verify the purity, impurity profiles, and key quality attributes of commercially sourced nobiletin, tectorigenin, hyperoside, and flavan-3-ols is a significant concern. This is because confounding effects from residual impurities cannot be excluded; these include notable tangeretin for nobiletin, potentially glycyrrhizic acid for tectorigenin sourced from Glycyrrhiza, co-existing flavonoids such as quercetin and rutin for hyperoside, and proanthocyanidins, which are common impurities in flavan-3-ol preparations. Consequently, it is difficult to reach a definitive conclusion that the observed bioactivities are attributable to the target metabolites alone.

Finally, silibinin (Li et al., 2020b; Wang et al., 2023) and baicalein (Li et al., 2022d) modified the gut microbiota, leading to favorable shifts in secondary metabolites like BAs and thereby mitigating NAFLD. However, silibinin and baicalein faced bioavailability limitations, a weakness circumvented by baicalein’s inhibition of breast cancer resistance protein (BCRP)/MRP2 transporters, which increase the absorption and bio-efficacy of silibinin (Xu et al., 2018). While preclinical studies in Caco-2 cells and mice suggest that baicalein inhibits BCRP/MRP2 to synergistically increase silybin bioavailability, critical human pharmacokinetic (PK) data are lacking (Li et al., 2020b). Crucially, the absence of human PK data means it is unknown if this botanical drug combination can achieve therapeutically effective concentrations of silybin in the human liver. Even as a mixture of silymarin and other flavonoid lignans, the clinical application value of silymarin is limited. In this 48-week, randomized, double-blind, placebo-controlled trial with biopsy-proven NASH, silymarin did not significantly improve hepatic inflammatory activity, but it showed potential in reducing liver fibrosis and improving relevant non-invasive markers, with a good safety profile (Wah Kheong et al., 2017). The inconsistent anti-inflammatory efficacy observed can likely be traced to two key limitations. One is the substantial batch-to-batch variability in silymarin content, which compromises dosing reproducibility. The other is the inherently poor aqueous solubility and low oral bioavailability of silymarin, which collectively diminish its pharmacological potency as a liver-targeted therapeutic.

While flavonoids show promise in ameliorating NAFLD by modulating BA metabolism, their translational value is undermined by critical deficiencies. These include their potential to act as pan-assay interference metabolites, unresolved mechanistic contradictions such as the tissue selectivity of FXR, the inherently low bioavailability of key metabolites, the use of preclinical models that fail to recapitulate human pathophysiology and ignore gender differences, and clinical studies limited by small scale, short duration, and a lack of histological endpoints. To advance the field, future research must employ more human-relevant models that include both sexes to resolve mechanistic ambiguities. It is also crucial to conduct structure-activity relationship studies and advance to large-scale, long-term clinical trials with standardized preparations and histological endpoints. Finally, developing innovative botanical drug delivery systems to enhance bioavailability and liver targeting is essential for realizing the therapeutic potential of flavonoids.

### 5.3 Saccharides

Oligosaccharides and polysaccharides from various sources, including plant-derived, tea-related, fungal, and marine-derived varieties, exhibit distinct therapeutic potentials in NAFLD management, with their efficacy correlating to structural complexity and multi-targeting capacity (Table 3).

**TABLE 3:** Saccharides for target BA metabolism in NAFLD.

Number	Agents	Dose	Experimental subject	Molecular targets	References
1	Stachyose	200 mg/kg/d	Male C57BL/6 J	FXR/SHP↓; CYP7A1, CYP27A1↑	Li et al. (2025)
2	Rhubarb polysaccharides	270, 540 mg/kg/d	Male C57BL/6	liver FXR, CYP7A1, BSEP↑; ileal FXR↓	Qiu et al. (2025)
3	Highland barley β-glucan	100 or 300 mg/kg/d	Male C57BL/6	liver FXR, CYP7A1, CYP27A1, CYP8B1↑; ileal FXR↓	Liu et al. (2022)
4	Raffinose	—	Male Wistar	12αOH BA↑	Maegawa et al. (2022)
5	Lycium barbarum oligosaccharide	200 mg/kg/d	Male C57BL/6 J	regulating intestinal flora	Li et al. (2023)
6	Astragalus polysaccharide	—	Male C57BL/6	CYP7A1↓; CYP7B1↑	Zheng et al. (2024)
7	Gracilaria lemaneiformis	—	Male Kunming	CYP7A1↑; LXRα↑; modulating *Bacteroides*	Huang et al. (2019)
8	Large-leaf yellow tea polysaccharides	100, 200, 400 mg/kg/d	Male C57BL/6	CYP7A1, CYP27A1, CYP7B1↑; CYP8B1↓; BSEP, NTCP↑; intestine FXR↓; liver FXR↑; ileum ASBT, I-BABP, OSTα/β↓; BSH,7α-dehydroxylated microbial species↓	Huang et al. (2024)
9	Ganoderma lucidum polysaccharide peptide	100 mg/kg/d	Male C57BL/6 J	CYP7A1, CYP8B1, FXR, SHP↑; SREBP-1c,FASN,ACC ↓	Zhong et al. (2018)

BA, bile acid; NAFLD, nonalcoholic fatty liver disease; FXR, farnesoid X receptor; SHP, small heterodimer partner; CYP7A1, cholesterol 7α-hydroxylase; CYP27A1, cholesterol 27α-hydroxylase; BSEP, bile salt export pump; CYP8B1, cytochrome P450 family 8 subfamily B polypeptide 1; LXRα, liver X receptor α; CYP7B1, cytochrome P450 family 7 subfamily B member 1; NTCP, sodium taurocholate co-transporting polypeptide; ASBT, apical sodium-dependent bile salt transporter; I-BABP, ileal BA, binding protein; OSTα/β, organic solute transporter α/β; BSH, bile salt hydrolases; SREBP1, sterol regulatory element-binding protein 1; FASN, fatty acid synthase; ACC, acetyl-coA, carboxylase.

A primary strategy involved the direct modulation of the enterohepatic FXR signaling axis to restore BA homeostasis. For instance, Stachyose ameliorated NAFLD by promoting hepatic synthesis of primary BAs by inhibiting the FXR/SHP pathway to upregulate CYP7A1 and CYP27A1, while simultaneously suppressing their gut microbial conversion into secondary BAs (Li et al., 2025). The stachyose study utilized a high-fat, high-sugar diet-induced model, which holds more clinical translational value as it recapitulates the metabolic and dysbiotic features of human NAFLD. Nevertheless, the investigation did not clarify if FXR inhibition was a direct effect or microbiota-dependent, nor did it assess the impact on intestinal barrier impairment associated with high sugar intake. Similarly, rhubarb polysaccharides and highland barley β-glucan both disrupted the intestinal FXR negative feedback loop to enhance hepatic BA synthesis. Rhubarb polysaccharides increased the expression of FXR, CYP7A1, and BSEP in the liver, while oppositely regulating intestinal FXR to prevent NAFLD (Qiu et al., 2025). However, the study was limited by its failure to elucidate the mechanism underlying this differential regulation and insufficient structural characterization of the polysaccharide. Highland barley β-glucan upregulated hepatic FXR, CYP7A1, CYP27A1, and CYP8B1 expression, but downregulated ileal FXR (Liu et al., 2022). The conclusions of this study were significantly undermined by using a 70% pure β-glucan preparation, leaving the 30% impurity fraction uncharacterized and its potential effects uninvestigated.

Beyond direct FXR modulation, many saccharides achieved their therapeutic effects indirectly by remodeling the gut microbiota, which in turn influenced BA metabolism. Raffinose reduced hepatic and systemic 12αOH BA levels yet did not lower cecal secondary 12αOH BA or transaminases, indicating its primary role in suppressing enterohepatic 12αOH BA recirculation to alleviate hepatic lipidosis (Maegawa et al., 2022). However, the model was limited to exogenous BA overload, omitting critical factors like obesity and insulin resistance. Lycium barbarum polysaccharide specifically regulated lipid metabolism to ameliorate hepatic steatosis (Xia et al., 2022), whereas its oligosaccharide counterparts appeared to function indirectly, as their steatosis reversal correlated with gut microbiota remodeling and BA metabolism modulation, suggesting gut-liver axis mediation (Li et al., 2023). The research on this astragalus polysaccharide took the regulation of intestinal flora as the core mechanism but failed to verify its causality through experiments such as FMT. Astragalus polysaccharide modulated BA metabolism, specifically by increasing the beneficial THDCA, which in turn downregulating CYP7A1 and regulating CYP7B1 to reduce hepatic fat accumulation (Zheng et al., 2024). Although this study clearly demonstrated that astragalus polysaccharides improved NAFLD by increasing THDCA, it did not verify whether the observed increase in THDCA levels was dependent on the prebiotic-like remodeling effect of the polysaccharides on the intestinal flora. Similarly, gracilaria lemaneiformis upregulated liver X receptor α (LXRα)/CYP7A1 and modulated *bacteroides*, demonstrating lipid-lowering and BA-conversion dual efficacy (Huang et al., 2019). The study was limited by the neglect of functional disparities in *bacteroides* between humans and mice and the unknown impurities remaining undefined.

Furthermore, certain polysaccharides exhibit sophisticated, organ-specific, multi-targeted synergistic actions. Large-leaf yellow tea polysaccharide (YTP) modulated BA synthesis by upregulating CYP7A1, CYP27A1, and CYP7B1 while inhibiting CYP8B1 (Huang et al., 2024). Notably, YTP exerted organ-specific FXR regulation, downregulating intestinal FXR but enhancing hepatic FXR expression, thereby coordinately elevating BSEP/NTCP-mediated BA efflux and suppressing ileal ASBT/I-BABP/OSTα/OSTβ to reduce cholesterol accumulation (Huang et al., 2024). Concurrently, YTP altered gut microbiota by depleting BSH- and 7α-dehydroxylation-active species, ultimately establishing gut-liver axis homeostasis as its core anti-NAFLD mechanism (Huang et al., 2024). A notable limitation was its exclusive use of male mice and a lack of detailed structural characterization, including key features like the degree of sulfation, methylation, and branching. Ganoderma lucidum polysaccharide peptide balanced BA synthesis and lipogenesis by upregulating CYP7A1, CYP8B1, FXR and SHP and inhibiting sterol regulatory element-binding protein 1c (SREBP-1c)/fatty acid synthase (FASN)/acetyl-coA carboxylase (ACC) (Zhong et al., 2018). This study was limited by its use of a single-gene knockout mouse model and the failure to validate the causality within the FXR-SHP pathway.

While existing evidence confirms the potential of oligosaccharides and polysaccharides to ameliorate NAFLD by modulating BA metabolism, their translational value is undermined by critical deficiencies. Key challenges include the use of unrepresentative preclinical models that fail to mimic core human NAFLD features, a frequent conflation of correlation with causation in mechanistic studies, and a lack of precise structural characterization that impedes clear structure-activity relationships. Furthermore, clinical evidence remains scarce, with large-scale, long-term randomized controlled trials (RCTs) using liver histology as the primary endpoint notably absent. To advance the field, future research must prioritize more clinically relevant animal models that recapitulate human pathology and employ rigorous methods, such as FMT and pathway inhibition, to establish true causality. It is also crucial to define precise structure-activity relationships by thoroughly characterizing the polysaccharides. Ultimately, these efforts must culminate in definitive clinical trials to validate efficacy and safety in human patients.

### 5.4 Saponins

Saponins collectively improve NAFLD through coordinated actions that regulate BA metabolism across the liver and intestines, modulate gut bacteria, and activate multi-target signaling pathways (Table 4).

**TABLE 4:** Saponins for target BA metabolism in NAFLD.

Number	Agents	Dose	Experimental subject	Molecular targets	References
1	Ginsenoside Rh4	60, 120, 180 mg/kg/d	Male C57BL/6 J	liver FXR, SHP↑; CYP7A1, CYP8B1↓; intestinal FXR, FGF15↓	Yang et al. (2023)
2	Betulinic acid derivative F6	3, 10, 30 mg/kg/d	Male C57BL/6	intestinal FXR↓; liver FXR↑	Zhang et al. (2022)
3	Ginsenoside Re	10, 20, 40 mg/kg/d	Male C57BL/6	liver FXR↑; CYP7A1↑	Zheng et al. (2025)
4	Saikosaponin D	10, 15 mg/kg/d	Male C57BL/6	intestinal FXR↓	Li et al. (2024b)
5	Astragaloside IV	12.5, 25, 50 mg/kg/d	Male C57BL/6 N	BSH↓; intestinal FXR↓; liver FXR↑	Zhai et al. (2022)
6	Cycloastragenol	—	Female C57BL/6	FXR↑	Gu et al. (2017)
7	Ilexsaponin A1	120 mg/kg/d	Male C57BL/6	CYP27A1, CYP7B1↑; liver FXR, BSEP↑; NTCP↓; BSH↑	Zhao et al. (2021)
8	Gypenoside A	1, 10, 100, 200 μg/mL	HepG2 cells	FXR↑; CYP7A1, CYP8B1↓; CYP27A1↑	Deng et al. (2024)
9	Glycyrrhizin	50 mg/kg/d	Male C57BL/6	FXR, SHP↑; CYP7A1↓	Yan et al. (2018a)
10	Soyasaponins A2	—	Male C57BL/6J (B6)	Intestinal FXR, FGF15↑	Xiong et al. (2021)

BA, bile acid; NAFLD, nonalcoholic fatty liver disease; FXR, farnesoid X receptor; SHP, small heterodimer partner; CYP7A1:cholesterol 7α-hydroxylase; CYP8B1, cytochrome P450 family 8 subfamily B polypeptide 1; FGF15, fibroblast growth factor 15; BSH, bile salt hydrolases; CYP27A1, cholesterol 27α-hydroxylase; CYP7B1, cytochrome P450 family 7 subfamily B member 1; BSEP, bile salt export pump; NTCP, sodium taurocholate co-transporting polypeptide.

A primary strategy was characterized by the dual modulation of FXR signaling, suppressing intestinal FXR to relieve its inhibition on hepatic BA synthesis, while simultaneously activating hepatic FXR to curb BA production and lipogenesis. For instance, ginsenoside Rh4 repaired the intestinal barrier and suppressed gut FXR/FGF15, while enhancing hepatic FXR/SHP signaling to inhibit BA synthesis (CYP7A1/CYP8B1) and lipogenic proteins like SREBP-1c and FASN (Yang et al., 2023). However, the NAFLD induction model in this study, which involved CCl_4_, presented a significant etiological discrepancy, potentially obscuring the specific efficacy of Rh4 against the metabolic pathology of human NAFLD. Betulinic acid derivative F6 selectively blocked intestinal FXR yet activated hepatic FXR to reduce harmful ceramides and inflammation, which ameliorated hepatic steatosis, inflammation, and fibrosis (Zhang et al., 2022). This study was limited by its exclusive use of male mice and a failure to elucidate the specific molecular trigger for hepatic FXR activation.

Building on FXR modulation, many saponins achieved this indirectly by remodeling the gut microbiota. Ginsenoside Re ameliorated NAFLD by modulating gut microbiota to upregulate hepatic FXR signaling, thereby inhibiting BA synthesis by upregulating CYP7A1 and reducing hepatic lipid accumulation, while concurrently repairing intestinal barrier and suppressing liver inflammation (Zheng et al., 2025). Similarly, saikosaponin D improved hepatic lipid deposition by remodeling of the gut microbiota, shifting BA profiles, and inhibiting intestinal FXR signaling (Li et al., 2024b). Astragaloside IV (AS-IV) also worked through the microbiome, modulating the intestinal flora to decrease BSH activity, thereby blocking harmful intestinal FXR signals, while activating protective FXR and GLP-1 pathways in the liver (Zhai et al., 2022). Notably, cycloastragenol, the active metabolite of AS-IV, directly bound to FXR to reduce liver triglycerides and enhance BA excretion (Gu et al., 2017). While promising, the translational value of these findings was often constrained by limitations such as the use of single-sex mouse models, a lack of validation for the causal involvement of the FXR pathway, and the failure to account for critical species differences in BA profiles.

In addition, some saponins targeted alternative BA synthesis and transport pathways. Ilexsaponin A1 ameliorated NAFLD by upregulating alternative BA synthesis CYP27A1/CYP7B1, enhancing hepatic efflux via FXR/BSEP, reducing NTCP-dependent reabsorption, and promoting fecal excretion via BSH activity, thereby lowering serum cholesterol, hepatic steatosis, and BA accumulation (Zhao et al., 2021). Gypenoside A, delivered via liposomes, also activated FXR to modulate key BA metabolic enzymes (such as inhibiting CYP7A1 and CYP8B1, upregulating CYP27A1) and correct the BA ratio imbalance (Deng et al., 2024). The translational relevance of the ilexsaponin A1 study was constrained by its use of only male mice, while the gypenoside A findings were limited to *in vitro* HepG2 cells without subsequent *in vivo* validation.

Finally, some saponins exhibited unique mechanisms. Glycyrrhizin reduced BA accumulation and partly ameliorated NASH not by directly enhancing FXR, but by restoring inflammation impaired FXR signaling, which in turn upregulated SHP and suppressed CYP7A1 (Yan et al., 2018a). Soyasaponin A2 increased ileal FXR/FGF15 signaling to enhance BA excretion in feces, while also rebalancing gut microbes (Xiong et al., 2021). This strategy, however, was constrained by its use of the MCD diet model and a lack of demonstrated causality between FXR activation and therapeutic efficacy.

In summary, saponins ameliorate NAFLD through a multi-targeted strategy centered on the enterohepatic BA circulation, with FXR signaling modulation being a key mechanism. This is achieved either through direct, organ-specific dual modulation of FXR, or indirectly via gut microbiota remodeling, complemented by actions on alternative BA pathways and the restoration of impaired FXR signaling. However, the translational potential of these findings is hampered by recurring methodological limitations. These include the use of NAFLD models that poorly reflect human disease etiology, a reliance on single-sex animal cohorts, and a frequent failure to establish causality between FXR modulation and therapeutic outcomes. To bridge this gap, future studies must employ more clinically relevant models in both sexes and incorporate rigorous causal validation, such as FXR knockout studies. Elucidating the molecular triggers for hepatic FXR activation and conducting comprehensive pharmacokinetic and safety assessments will be essential to advance promising saponin candidates toward clinical application.

### 5.5 Alkaloids

Another major class of natural products with potent anti-NAFLD effects is the alkaloids. This diverse group includes metabolites such as the aporphine alkaloid nuciferine and the well-studied isoquinoline alkaloid berberine (Table 5).

**TABLE 5:** Alkaloids for target BA metabolism in NAFLD.

Number	Agents	Dose	Experimental subject	Molecular targets	References
1	Nuciferine	10, 25 mg/kg/d	Male SD	Ileal FXR, ASBT, I-BABP, OSTα/β↓; CYP7A1, CYP27A1↑; BSH-producing and 7α-dehydroxylating genera↓	Sun et al. (2022)
2	Berberine	100 mg/kg/d	Male LVG Syrian hamsters	Regulating intestinal flora	Gu et al. (2015)
3	Berberine	50 mg/kg/d	Male and female descendants of C57Bl/6J and 129S1/SvlmJ (B6/129)	CYP27A1, CYP8B1, CYP7A1, CYP7B1, SHP, FXR, NTCP↑	Wang et al. (2021b)
4	Berberine	1500 mg/d	Human	—	Koperska et al. (2024)
5	Berberine	1500 mg/d	Human	—	Yan et al. (2015)

BA, bile acid; NAFLD, nonalcoholic fatty liver disease; SD, Sprague-Dawley; FXR, farnesoid X receptor; ASBT, apical sodium-dependent bile salt transporter; I-BABP, ileal BA, binding protein; OSTα/β, organic solute transporter α/β; CYP7A1, cholesterol 7α-hydroxylase; CYP27A1, cholesterol 27α-hydroxylase; BSH, bile salt hydrolases; LVG, lakeview golden; CYP8B1, cytochrome P450 family 8 subfamily B polypeptide 1; CYP7B1, cytochrome P450 family 7 subfamily B member 1; SHP, small heterodimer partner; NTCP, sodium taurocholate co-transporting polypeptide.

A key therapeutic approach involved the direct, coordinated modulation of the enterohepatic axis and the gut microbiota to correct BA dysregulation. For instance, the aporphine alkaloid nuciferine reduced hepatic lipid accumulation and injury by targeting both the enterohepatic axis and the gut microbiota. It downregulated ileal FXR and associated transporters (ASBT, I-BABP, OSTα/β) while upregulating hepatic BA synthesis enzymes (CYP7A1, CYP27A1), alongside suppressing BSH-producing and 7α-dehydroxylating bacteria to reshape gut microbiota (Sun et al., 2022). However, the limitation to male rats precluded an assessment of sex-based generalizability. Moreover, the proposed regulatory effect of the microbiota on BA deconjugation was not directly confirmed, as fecal BSH activity was not measured.

Building on this, orally administered berberine achieved its therapeutic effects indirectly by first reshaping the gut microbiota, which in turn modulates host BA signaling pathways. Due to low bioavailability, berberine accumulated in the gut and ameliorated hyperlipidemia by increasing the total BA pool, an effect linked to a higher Firmicutes/Bacteroidetes ratio and inhibited *clostridium* activity (Gu et al., 2015). While the research provided a valuable pharmacokinetic comparison between oral and intraperitoneal routes to explain its efficacy despite low plasma levels, it was limited by the lack of intestinal FXR pathway evaluation and dose-group variations. In a separate study, berberine restored BA homeostasis through upregulating key enzymes in BA synthesis (CYP27A1, CYP8B1, CYP7A1 and CYP7B1), nuclear receptors (SHP and FXR), and the liver-specific transporters NTCP, thereby reducing hyperlipidemia (Wang et al., 2021b). While the inclusion of both male and female mice prevented gender bias and it proposed a mechanism involving gut microbiota modulation by berberine, its conclusions were weakened by the lack of direct microbial analysis, the absence of FMT to establish the microbiota’s mediating role in BA metabolism, and the omission of a dose-response assessment.

The therapeutic potential of berberine has also been explored in clinical settings, though with distinct challenges. A 12-week randomized controlled trial demonstrates that a daily dose of 1,500 mg berberine significantly improves liver enzymes and cholesterol levels in patients with MAFLD (Koperska et al., 2024). While the robust, double-blind, placebo-controlled design and 12-week follow-up confirmed its safety, the findings were limited by a significant baseline disparity between groups, the lack of direct imaging for hepatic fat, and a small sample size. Furthermore, a study on berberine combined with lifestyle intervention showed it effectively treated NAFLD by reducing hepatic fat and body weight, benefits driven by its high hepatic bioavailability and good safety profile (Yan et al., 2015). Key limitations of this study persist, including the lack of histological assessment for inflammation and fibrosis, an undefined role for gut microbiota, and the exclusion of female subjects from the clinical trial.

In summary, diverse alkaloids, including nuciferine and berberine, ameliorated NAFLD through a multi-targeted strategy that centered on the enterohepatic BA circulation and gut microbiota. Their mechanisms ranged from the direct, dual modulation of intestinal and hepatic FXR signaling to indirect actions that were mediated by profound remodeling of the gut microbiota. While preclinical evidence was compelling and early clinical trials were promising, the translational potential of these findings was consistently hampered by recurring methodological limitations. These included the frequent use of single-sex animal cohorts, a failure to establish causality between microbiota changes and host outcomes, and a lack of comprehensive clinical data such as histology and imaging. To bridge this gap, future research have to prioritize studies in both sexes, incorporate rigorous causal validation techniques like FMT or pathway knockouts, and design more robust clinical trials with appropriate histological and imaging endpoints to fully validate the therapeutic promise of these natural agents.

### 5.6 Curcuminoids

The therapeutic potential of curcumin and its derivatives in NAFLD has been extensively investigated, revealing a multi-faceted approach to restoring BA homeostasis (Table 6).

**TABLE 6:** Curcuminoids for target BA metabolism in NAFLD.

Number	Agents	Dose	Experimental subject	Molecular targets	References
1	Curcumin	50, 100 mg/kg/d	Male C57BL/6	FXR, CYP7A1↑	Yan et al. (2018b)
2	Tetrahydrocurcumin	100 mg/kg/d	Male C57BL/6	MRP2, BSEP↑; regulating intestinal flora	Peng et al. (2025)
3	Curcumin	500 mg/d	Human	regulating intestinal flora	He et al. (2024b)
4	Phospholipid curcumin	250 mg/d	Human	—	Chashmniam et al. (2019)
5	Phospholipid curcumin meriva	2000 mg/d	Human	—	Musso et al. (2025)

BA, bile acid; NAFLD, nonalcoholic fatty liver disease; FXR:farnesoid X receptor; CYP7A1, cholesterol 7α-hydroxylase; MRP2, multidrug resistance-associated protein 2; BSEP, bile salt export pump.

Initial preclinical work demonstrated that curcumin ameliorated hepatic steatosis by upregulating the expression of hepatic FXR and CYP7A1 and inhibiting the activity of LXRα (Yan et al., 2018b). However, the foundational study was limited by its exclusive use of male mice and a lack of focus on specific BA species or the enterohepatic circulation. Building on this, its major metabolite, tetrahydrocurcumin ameliorated NAFLD by reducing serum levels of toxic BAs, such as 7-keto-deoxycholic acid (7-KDCA) and CA, upregulating hepatic BA efflux transporters, including MRP2 and BSEP, decreasing the Firmicutes phylum, and increasing the Verrucomicrobiota phylum (Peng et al., 2025). This suggested a synergistic gut-liver axis effect, yet the study was constrained by its failure to verify the role of BA receptors or explore the microbiota’s influence on BA-transforming enzymes.

This gut-centric mechanism was further validated in human subjects. A randomized controlled trial found that curcumin exerted anti-NAFL effects by modulating the gut microbiota, reducing the Firmicutes/Bacteroidetes ratio, increasing *Bacteroides* abundance, and enhancing fecal BSH activity to promote BA deconjugation and secondary BA generation (He et al., 2024b). Nevertheless, this clinical proof-of-concept was limited by its use of a single dose and a patient cohort restricted to early-stage NAFL from a single center. To enhance clinical translational potential, formulation strategies were explored. In a randomized, double-blind, clinical trial, phospholipid curcumin ameliorated NAFLD by reducing serum BAs (such as CDCA, TCA, and LCA) (Chashmniam et al., 2019). Nevertheless, the study was constrained by its small sample size and the absence of liver biopsy-confirmed pathology. More definitive clinical evidence came from a subsequent efficacy trial in patients with biopsy-proven NASH. Phospholipid curcumin meriva achieved significant NASH resolution and fibrosis improvement with good long-term (72-week) safety (Musso et al., 2025). This provided key clinical evidence for the formulation, but the study was limited by a small sample size, the absence of dose-response data, the exclusion of F4 cirrhosis patients, and a lack of focus on BA mechanisms.

In summary, the evidence indicated that curcumin and its derivatives, particularly in advanced formulations, ameliorated NAFLD through a multi-faceted approach targeting the enterohepatic BA axis and gut microbiota. The mechanistic understanding evolved from direct hepatic receptor modulation to a broader model involving gut microbiota remodeling and enhanced BA excretion. This progression was paralleled by a translational path from preclinical models to human trials, ultimately leading to a study that demonstrated histological improvement in biopsy-proven NASH patients. Despite this progress, the research body was consistently hampered by recurring limitations, including single-sex animal studies, a lack of causal validation for microbiota effects, and clinical constraints such as small sample sizes and insufficient mechanistic endpoints. To fully validate their therapeutic potential, future investigations must integrate comprehensive BA profiling, establish causality between microbiota shifts and host outcomes, and conduct larger, multi-center clinical trials with robust histological endpoints.

### 5.7 Lignans

Another major class of natural products with potent anti-NAFLD effects was the lignans, which demonstrated significant therapeutic potential by targeting the enterohepatic axis and BA metabolism (Table 7).

**TABLE 7:** Lignans and iridoid glycosides for target BA metabolism in NAFLD.

Category	Agents	Dose	Experimental subject	Molecular targets	References
Lignans	Honokiol	20 mg/kg/d	male C57BL/6	OATP-1B2↑; gut probiotics↑; harmful bacteria↓	Zhai et al. (2023)
	Deoxyschizandrin	25 μM; 50 mg/kg	HepG2, L02 cells; female C57BL/6 J	Liver and central nervous system FXR/TGR5 signaling↑	Gu et al. (2023)
	Schisandra chinensis lignans	25, 50, 80 μg/mL 20, 40, 80 μg/mL	HepG2 cells; male C57BL/6 J	Liver and intestinal FXR/TGR5 signaling↑	Yan et al. (2025)
Iridoid glycosides	Gentiopicroside	50, 100 mg/kg/d	malec57bl/6J	FXR↑; CYP7A1, SREBP1, FASN↓	Li et al. (2024c)

BA, bile acid; NAFLD, nonalcoholic fatty liver disease; OATP-1B2, organic anion transporting polypeptide 1B2; FXR, farnesoid X receptor; TGR5, G-protein coupled bile acid receptor 1; CYP7A1, cholesterol 7α-hydroxylase; SREBP1, sterol regulatory element-binding protein 1; FASN, fatty acid synthase.

A key therapeutic approach involved the direct modulation of hepatic transporters and the gut microbiota to correct serum BA dysregulation. Honokiol was found to correct serum BA dysregulation by reducing 23-DCA and TDCA, potentially via upregulating hepatic OATP-1B2 and modulating the gut microbiota (increasing Ruminococcaceae, decreasing Firmicutes) (Zhai et al., 2023). The study was limited by a lack of direct measurement of key intestinal BA metabolic genes and the absence of FMT to confirm a causal link.

Building on this, other lignans targeted key nuclear receptors within the enterohepatic axis. Deoxyschizandrin improved NAFLD through activating hepatic and central nervous system FXR/TGR5 signaling and enhancing leptin sensitivity (Gu et al., 2023). However, the target specificity was not verified in gene knockout mice, and the efficacy was not compared with that of known FXR/TGR5 agonists. Similarly, schisandra chinensis lignans ameliorated NASH by activating the enterohepatic FXR/FGF15 signaling axis, modulating the gut microbiota, reducing hepatic BA accumulation, and promoting fecal BA excretion (Yan et al., 2025). While the study precisely defined the composition of the Schisandra lignan mixture and quantified the proportion of each metabolite, it failed to identify the specific lignans responsible for the core regulatory effects on FXR and BA metabolism, nor did it validate the causal role of the gut microbiota through FMT.

In summary, lignans such as honokiol and deoxyschizandrin ameliorated NAFLD by targeting the enterohepatic BA circulation and gut microbiota. Their mechanisms included upregulating hepatic transporters and activating nuclear receptors like FXR and TGR5. However, their translational potential was hampered by methodological limitations, including a failure to establish causality for microbiota changes, a lack of target specificity, and incomplete identification of active metabolites. Future research must therefore prioritize causal validation, direct comparisons with established agonists, and chemical fractionation to fully validate their therapeutic promise.

### 5.8 Iridoid glycosides

Iridoid gentiopicroside rectified gut dysbiosis and reduced hepatic fat via FXR upregulation and suppression of CYP7A1/sterol regulatory element-binding protein 1 (SREBP1)/FASN (Li et al., 2024c). The use of a western diet and CCl_4_ model that closely resembled human lean NASH was a notable strength of the study, yet the investigation was limited by its male-only cohort and its failure to experimentally validate the role of the microbiota through direct fecal BA analysis or by employing FMT to prove causality (Table 7).

### 5.9 Sterols/terpenoids

Another major class of natural products with potent anti-NAFLD effects comprised sterols, triterpenes, and diterpenes. A key therapeutic approach for these metabolites involved the coordinated modulation of the enterohepatic axis and the gut microbiota to correct BA dysregulation (Table 8).

**TABLE 8:** Sterols and terpenoids for target BA metabolism in NAFLD.

Number	Agents	Dose	Experimental subject	Molecular targets	References
1	Stigmasterol	200 mg/kg/d	Male C57BL/6 J	CYP7B1↑	Xin et al. (2023)
2	Diosgenin	150, 300, 400 mg/kg/d	Male SD	regulating intestinal flora	Zhou et al. (2022)
3	Diosgenin	30 mg/kg, every other day	Male C57BL/6 J	liver FXR/SHP, intestinal FXR/FGF15↑, CYP7A1↓	Yan et al. (2023)
4	Diosgenin	30 mg/kg, every other day	Male C57BL/6 J	CYP7A1 ↓	Yan et al. (2024)
5	Celastrol	100, 200, 300 μg/kg/d	Male C57BL/6 J	FXR/LXR, CYP7B1↑	Hu et al. (2025)
6	Acanthoic acid	5 μM; 20, 40 mg/kg/d	AML-12 cells; male C57BL/6	FXR↑	Han et al. (2019)

BA, bile acid; NAFLD, nonalcoholic fatty liver disease; CYP7B1, cytochrome P450 family 7 subfamily B member 1; SD, SD, Sprague-Dawley; FXR, farnesoid X receptor; SHP, small heterodimer partner; FGF15, fibroblast growth factor 15; CYP7A1, cholesterol 7α-hydroxylase; LXRα, liver X receptor; BSEP, bile salt export pump.

One study reported that enhancing the alternative BA synthesis pathway was a key mechanism. Stigmasterol enhanced this pathway via CYP7B1, converting cholesterol into more hydrophilic BAs for excretion, an effect coupled with increased gut microbial diversity (Xin et al., 2023). However, the study failed to validate the necessity of the CYP7B1 enzyme through knockout models, nor did it confirm the causal impact of microbiota alterations via FMT.

Research on diosgenin revealed a more complex, multi-faceted mechanism. One study demonstrated that diosgenin reduced hepatic lipid accumulation in rats with NAFLD by decreasing the abundance of specific gut microbiota (Globicatella and Phascolarctobacterium) and improving fecal BA metabolism (Zhou et al., 2022). However, it used only male animals, employed a simple HFD model, and did not validate the causal role of the microbiota or metabolites through interventions like FMT or metabolite supplementation. A subsequent study provided deeper mechanistic insight, showing that diosgenin alleviated NASH by increasing the abundance of Clostridia and fecal BSH activity to modulate BA metabolism, while simultaneously activating hepatic FXR/SHP and intestinal FXR/FGF15 signaling to suppress CYP7A1 (Yan et al., 2023). Notably, while the mechanism was more detailed, the findings were derived from male mice using an MCD diet, which did not fully recapitulate human NAFLD pathogenesis. This was further refined by another report, diosgenin downregulated CYP7A1 expression in a concentration-dependent manner, thereby affecting BAs such as CDCA, CA, and TCA in the liver and feces (Yan et al., 2024). However, the study was limited to male mice and did not investigate whether the modulation of BA metabolism by the gut microbiota contributed to the therapeutic efficacy of diosgenin.

Similarly, celastrol and acanthoic acid were investigated for their potent effects on BA signaling, though with distinct mechanistic focuses and limitations. Celastrol ameliorated cholestasis by activating the FXR/LXR signaling axis, leading to the upregulation of CYP7B1 and a correction of the toxic/non-toxic BA imbalance (Hu et al., 2025). However, its investigation was confined to male mice and overlooked the potential contribution of the gut microbiota. In a parallel line of inquiry, acanthoic acid activated FXR and LXRs, suggesting the FXR-LXR axis is an effective target for NAFLD (Han et al., 2019). Nevertheless, the study was limited by the exclusion of female mice, a lack of direct quantification of BA species, and the absence of validation for key downstream enzymes and transporters.

In summary, sterols, triterpenes, and diterpenes such as stigmasterol, diosgenin, celastrol, and acanthoic acid, ameliorated NAFLD primarily by targeting the enterohepatic circulation of BAs. Their therapeutic strategies evolved from observations of enhanced BA excretion and microbiota shifts to more sophisticated models involving the direct activation of key nuclear receptors like FXR and LXR. The research on diosgenin, showcased a progression from a microbiota-centric effect to a direct, receptor-mediated mechanism, highlighting the complexity of their actions. However, the translational potential of this entire class of metabolites was consistently hampered by recurring methodological limitations. These included a pervasive reliance on single-sex animal cohorts, a frequent failure to establish causality between microbiota changes and host metabolic outcomes, and a lack of comprehensive validation for proposed molecular pathways. To fully harness their therapeutic promise, future investigations have to prioritize studies in both sexes, incorporate rigorous causal validation techniques like FMT or genetic knockouts, and ensure that proposed mechanisms were supported by direct biochemical evidence.

### 5.10 Phenolic acids/other phenolics

Another major class of natural products, phenolic metabolites, demonstrated potent anti-NAFLD effects, primarily through sophisticated modulation of the enterohepatic axis and gut microbiota to correct BA dysregulation. A key therapeutic strategy for these metabolites involved targeting the FXR signaling pathway, albeit with distinct upstream mechanisms (Table 9).

**TABLE 9:** Phenolic acids and other phenolics for target BA metabolism in NAFLD.

Number	Agents	Dose	Experimental subject	Molecular targets	References
1	Salidroside	50 mg/kg/d	Female C57BL/6J	liver FXR ↑	Zhan et al. (2022)
2	Tyrosol	23 mg/kg/d	Female C57BL/6J	liver FXR ↑	Zhan et al. (2022)
3	Hydroxytyrosol	26 mg/kg/d	Female C57BL/6J	liver FXR ↑	Zhan et al. (2022)
4	Salidroside	20 mg/kg/d	Male C57BL/6	liver and ileal FXR ↑	Li et al. (2020c)
5	Salidroside	25, 50 mg/kg/d	Male C57BL/6J	FXR/FGF15 ↑; regulating intestinal flora; BSH ↑	Zhang et al. (2025)
6	Rhubarb anthraquinone	37.5, 75, 150 mg/kg	Male SD	—	Zhang et al. (2024b)
7	Ellagic acid	100 mg/kg/d	Male C57BL/6	regulating intestinal flora	Luo et al. (2025)
8	Ellagic acid	180 mg	Human	—	Mighani et al. (2025)

BA, bile acid; NAFLD, nonalcoholic fatty liver disease; FXR, farnesoid X receptor; FGF15, fibroblast growth factor 15; SD, Sprague-Dawley.

For instance, several phenolics, salidroside, tyrosol, and hydroxytyrosol were shown to attenuate hepatic steatosis by upregulating hepatic FXR, maintaining BA homeostasis, and modulating the gut microbiota (Zhan et al., 2022). Among them, salidroside has been the subject of particularly detailed investigation, revealing multiple, nuanced mechanisms of action. One study demonstrated that salidroside ameliorated NASH by modulating the gut microbiota, which rectified BA metabolism by decreasing colonic toxic/conjugated BAs (such as DCA, TCA and tauro-α/β-muricholic acid (Tα/β-MCA)) and increasing β-CDCA, ultimately activating the hepatic and ileal FXR signaling axis, as well as the ileal FGF15 signaling axis (Li et al., 2020c). Nevertheless, the investigation was restricted to male mice and failed to establish whether the FXR/FGF15 pathway was essential for the observed therapeutic effects. A complementary mechanism for salidroside was proposed in another study, which found that it ameliorated NASH by upregulating the abundance of Bacteroidota and *Bacteroides* in the gut microbiota, enhancing fecal BSH activity, reducing hepatic levels of conjugated BAs, such as Tα/β-MCA, and activating FXR and TGR5 (Zhang et al., 2025). While the study rigorously established this molecular mechanism through FMT, antibiotic intervention, and inhibitor experiments, it was limited to male mice and did not explore the specific molecular mechanism by which *Bacteroides* regulates BSH activity.

Beyond salidroside, other phenolics act through different pathways. Rhubarb anthraquinone, for example, intervened in primary BA biosynthesis, leading to reduced hepatic levels of specific abnormal BAs, such as the toxic DCA and the conjugated TCA (Zhang et al., 2024b). However, this study did not explore the specific mechanisms of enzyme and transporter modulation, nor the involvement of BA receptors.

Similarly, ellagic acid ameliorated NAFLD by reducing the abundance of gut bacteria associated with impaired barrier function like Alistipes, Colidextribacter, and Ruminococcus, enriching the beneficial Faecalibaculum, and correcting hepatic metabolic disorders through the upregulation of TDCA and TCDCA (Luo et al., 2025). Nevertheless, this work was also conducted exclusively in male mice and lacked verification of BA receptor involvement or the role of microbial metabolites. Crucially, the therapeutic potential of ellagic acid was supported by a randomized, double-blind, placebo-controlled clinical trial. This well-controlled study demonstrated that daily supplementation with 180 mg of ellagic acid for 8 weeks significantly ameliorated liver enzymes, triglycerides, insulin resistance, and pathological markers of oxidative stress and inflammation in NAFLD patients (Mighani et al., 2025). Despite this promising clinical translation, the study was limited by a small sample size, a short intervention duration, and the absence of imaging-based assessment of liver fat.

In summary, while phenolic metabolites consistently correct BA dysregulation to ameliorate NAFLD, the translational potential of these findings is currently hampered by significant methodological limitations. A pervasive reliance on single-sex animal cohorts, a frequent failure to establish causality between microbiota changes and host outcomes, and a lack of comprehensive validation for proposed molecular pathways are common shortcomings. To fully harness their therapeutic promise, future investigations must prioritize studies in both sexes, incorporate rigorous causal validation techniques like FMT or genetic knockouts, and ensure that proposed mechanisms are supported by direct biochemical evidence.

## 6 Comparative analysis of natural products and existing pharmacotherapies

The therapeutic pipeline for NAFLD/NASH explored diverse targets and achieved a landmark breakthrough with the approval of the first drugs. For instance, the GLP-1 receptor agonist semaglutide, while significantly ameliorating hepatic inflammation and steatosis, was limited in its clinical application by a high incidence of gastrointestinal side effects and limited antifibrotic efficacy in the overall population (Sanyal et al., 2025). Similarly, THR-β agonist resmetirom, which demonstrated efficacy in inducing NASH resolution and fibrosis improvement, was associated with an increased risk of gastrointestinal side effects such as diarrhea and nausea (Harrison et al., 2024). These findings indicated that even these breakthrough drugs were not universal solutions, underscoring the persistent need for therapies with broader efficacy and better tolerability.

Notably, among these approved and investigational drugs, the modulation of BA metabolism remained a core therapeutic strategy. However, the representative drug for this pathway, the FXR agonist OCA, while improving hepatic pathology by activating the FXR pathway, was restricted in its long-term application by significant side effects such as pruritus (Younossi et al., 2019). In stark contrast, the ten classes of natural products, which also exerted their effects by modulating BA metabolism, demonstrated distinct advantages in mechanistic diversity, safety, tolerability, and spectrum of efficacy due to their multi-target properties. This positioned them as important complementary or alternative options to existing Western medicines.

From a mechanistic perspective, Western drugs predominantly relied on a single target as their primary mechanism of action (next-generation FXR agonists like vonafexor/tropifexor or OCA primarily activated the FXR pathway). This focused strategy often struggled to ameliorate the multiple pathological links of NAFLD systemically and simultaneously, the BA dysbiosis-gut microbiota imbalance inflammation/fibrosis axis, in the way that natural products did through multi-target synergy. In terms of efficacy, single-target Western drugs showed a limited therapeutic spectrum: the primary efficacy of OCA was demonstrated in fibrosis improvement, whereas its effects on steatosis or ballooning degeneration did not reach statistical significance, and rebound effects were observed after discontinuation. Natural products, conversely, achieved broad-spectrum efficacy; for example, berberine and Astragalus polysaccharides not only improved liver enzymes and reduced steatosis but also possessed multiple functions including anti-inflammatory, anti-fibrotic, and gut microbiota-modulating effects.

Regarding safety, the significant side effects of Western drugs often restricted their long-term use. For OCA, the incidences of FXR activation-induced pruritus severely limited patient adherence. Natural products, by contrast, generally exhibited superior safety profiles, owing to their multi-target, low-intensity, gentle modulating properties and the favorable data reported in clinical studies. For example, berberine at 1,500 mg/day for 12 weeks caused no serious adverse reactions (Koperska et al., 2024), ellagic acid at 180 mg/day for 8 weeks did not induce gastrointestinal discomfort (Mighani et al., 2025), and the phytosomal curcumin formulation meriva was even safely administered for a long-term course of 72 weeks (Musso et al., 2025). Furthermore, most natural products (quercetin, Qianggan capsules) lacked metabolism-related side effects, making them more suitable for the long-term management of NAFLD.

## 7 Conclusions and prospects

This review summarized the potential of natural products including TCM formulas, flavonoids, saccharides, saponins, alkaloids, curcuminoids, lignans, iridoid glycosides, sterols/terpenoids and phenolic acids/other phenolics to treat NAFLD by regulating the BA metabolic network. Despite their significant efficacy in preclinical models, the application of natural products faces challenges such as low bioavailability, variability due to metabolite complexity, differences in tissue-specific effects, and potential side effects.

### 7.1 Bioavailability issues

Low oral bioavailability is a pervasive challenge for natural products, stemming from their inherent structural properties or physiological barriers, with distinct manifestations across different classes. Lipophilic metabolites (silymarin) suffer from poor aqueous solubility. Acid-labile molecules (EGCG, curcuminoids) are prone to degradation in the gastrointestinal tract. Furthermore, certain metabolites (berberine) exhibit limited hepatic targeting due to intestinal sequestration. Complex systems like TCM formulas and saccharides (barley β-glucan) also face bioavailability issues due to their intricate molecular structures or insufficient purity.

Future research should prioritize advanced delivery technologies. For lipophilic metabolites, nanocarriers or lipid-based formulations can enhance solubility and hepatic targeting. Enteric coating can be designed for acid-sensitive metabolites to resist degradation. Additionally, investigating the synergistic effects of metabolite compatibility (such as the combination of berberine with flavonoids (Miskolczy et al., 2016)) is another strategy. For complex systems like TCM formulas, enriching active metabolite clusters through fractionation or adopting modular delivery strategies represents a viable approach to improve bioavailability.

### 7.2 Metabolite variation and quality control

The heterogeneity of metabolites severely compromises the reproducibility of therapeutic effects for various natural products. The synergistic action of TCM formulas is highly dependent on the source of raw materials and extraction protocols, yet the core active substances in most formulas remain unidentified. Within single-metabolite classes, samples often contain uncharacterized impurities, while batch-to-batch purity variations confound the attribution of bioactivity. Mixtures such as saccharides (ganoderma lucidum polysaccharide peptides) and lignans (schisandra mixtures) lack fine structural characterization (such degree of branching, sulfation) or have failed to identify the core metabolites responsible for modulating BA metabolism.

Therefore, standardization protocols must be both comprehensive and adaptable. For complex systems (TCM formulas, mixed extracts), it is crucial to identify core active biomarkers directly linked to BA regulation and establish quality control systems based on fingerprint chromatography. For single-metabolite classes, high-purity standards should be enforced through advanced purification techniques or synthetic biology. For complex molecules (saccharides, lignans), techniques like NMR and mass spectrometry should be employed to elucidate the key structural features that influence BA-regulatory activity.

### 7.3 Multi-metabolite interactions and mechanistic heterogeneity

Each class of natural product exhibits unique mechanisms in regulating BA metabolism, leading to seemingly paradoxical phenomena that necessitate a systems pharmacology approach for clarification. These contradictions primarily arise from the inherent complexity of these natural products and limitations in experimental design. The heterogeneity in FXR modulation is evident in TCM formulas (LG activating intestinal FXR while Zhuyu Pills inhibit it), flavonoids (isoquercitrin inhibiting intestinal FXR while quercetin activates hepatic FXR), and the dual regulation by saponins (ginsenoside Rh4). This complexity is often a direct consequence of their multi-metabolite, multi-target nature, where various metabolites can exert synergistic or antagonistic effects on the same pathway, leading to complex regulatory outcomes. Differences in microbiota-BA interactions are observed with alkaloids (berberine) and phenolic acids (salidroside). Saccharides (astragalus polysaccharide) and sterols/terpenoids (diosgenin) may act through both direct regulation of BA enzymes and indirect effects on the microbiota. Pathway diversity is also significant; for instance, lignans (deoxyschisandrin) integrate FXR/TGR5 activation with leptin sensitivity regulation. Furthermore, variations in experimental models, including differences in animal strains and modeling methods, also contribute to these divergent observations. Notably, most of these findings remain at the correlative level, lacking causal validation, which makes it difficult to distinguish whether the observed effects are due to the holistic action of the formula or the direct action of specific metabolites.

Resolving these complexities requires a systematic approach that involves integrating multi-omics technologies (transcriptomics, metabolomics, metagenomics) to map the metabolite-target-BA phenotype network, and employing causal validation tools (such as FXR knockout models, FMT) to distinguish direct effects from microbiota-mediated indirect effects.

### 7.4 Safety and dose rationality

The rationale for preclinical dosing is often unclear in two key aspects. First, the justification for dose selection is frequently absent. Many studies do not clarify whether doses are based on toxicity testing, literature references, or arbitrary choices, leading to inconsistent dosing that hinders cross-study comparisons. Second, the relevance to human equivalent doses (HED) is often overlooked. Few studies mention the use of standardized methods (the Reagan-Shaw equation) for dose conversion. Inter-species differences in BA metabolism, such as higher CYP7A1 activity in mice than in humans or variations in specific gut microbiota (Akkermansia), suggest that an effective preclinical dose may not translate to human efficacy.

Furthermore, there is a critical lack of long-term safety data for most classes of natural products intended for NAFLD therapy. Most categories, including TCM formulas, flavonoids, saponins, and alkaloids, lack systematic long-term toxicological data. For instance, it is unknown whether long-term intervention with metabolites known to modulate key metabolic enzymes or nuclear receptors disrupts endogenous metabolic homeostasis. Similarly, the long-term impact of microbiota-altering substances on gut barrier function lacks clinical evidence.

Future research must establish clear dose-response relationships. Preclinical studies should test multiple doses to define a therapeutic window (monitoring serum ALT/BA levels to assess toxicity). Standardized HED conversion should be mandatory, using validated formulas to correlate preclinical and human doses. Finally, prioritizing humanized models, such as humanized microbiota mice or patient-derived liver organoids, is essential for categories highly dependent on the microbiota-BA axis to reduce inter-species bias.

### 7.5 Inconsistent clinical evidence

The strength of clinical validation varies significantly among different classes. Categories with relatively robust evidence include curcuminoids (phospholipid curcumin meriva), which have shown NASH resolution in biopsy-proven trials; alkaloids (berberine), which have improved liver enzymes in small-scale RCTs; and phenolic acids (ellagic acid), which have ameliorated insulin resistance in NAFLD patients. In contrast, evidence for most other categories is weak or absent. Catechins (EGCG) lack placebo-controlled trials; TCM formulas (Ling-Gui-Zhu-Gan decoction) have only preliminary human data on improving insulin resistance; and lignans, iridoid glycosides, and most saccharides have no large-scale clinical data to date.

Future clinical trials must be designed as large-scale, multi-center RCTs with liver histology as the primary endpoint, incorporating long-term follow-up to assess safety.

In conclusion, natural products, by virtue of their multi-target regulation of the BA metabolic network, present a promising avenue for the treatment of NAFLD. However, translating this potential into clinical practice requires overcoming critical hurdles in bioavailability, quality control, mechanistic clarity, safety, and clinical validation. Future efforts must focus on developing innovative delivery systems, establishing rigorous quality standards, employing systems-level approaches to elucidate complex mechanisms, and conducting well-designed clinical trials. By addressing these challenges and leveraging emerging technologies, we can accelerate the translation of these therapies from the bench to the bedside, ultimately equipping patients with more effective and safer personalized treatment options.

## Glossary

NAFLD: nonalcoholic fatty liver disease
NASH: nonalcoholic steatohepatitis
HCC: hepatocellular carcinoma
NAFL: nonalcoholic fatty liver
BA: bile acid
MAFLD: metabolic dysfunction-associated fatty liver disease
OCA: Obeticholic acid
FXR: farnesoid X receptor
THR-β: thyroid hormone receptor-β
TCM: traditional Chinese medicine
CYP7A1: cholesterol 7α-hydroxylase
C4: 7α-hydroxy-4-cholesten-3-one
CA: cholic acid
CYP8B1: cytochrome P450 family 8 subfamily B member 1
CDCA: chenodeoxycholic acid
CYP27A1: cholesterol 27α-hydroxylase
CYP7B1: cytochrome P450 family 7 subfamily B member 1
GCA: glycocholic acid
GCDCA: glycochenodeoxycholic acid
TCA: taurocholic acid
TCDCA: taurochenodeoxycholic acid
BSH: bile salt hydrolases
DCA: deoxycholic acid
LCA: lithocholic acid
UDCA: ursodexoycholic acid
GDCA: glycodeoxycholic acid
TDCA: taurodeoxycholic acid
GLCA: glycolithocholic acid
TLCA: taurolithocholic acid
GUDCA: glycoursodeoxycholic acid
TUDCA: tauroursodeoxycholic acid
BSEP: bile salt export pump
MRP: multidrug resistance-associated protein
OST: organic solute transporter
ASBT: apical sodium-dependent bile salt transporter
I-BABP: ileal BA binding protein
OATPs: organic anion-transporting polypeptides
NTCP: sodium taurocholate co-transporting polypeptide
OATPs: organic anion-transporting polypeptides
SHP: small heterodimer partner
HNF4α: hepatocyte nuclear factor 4α
BARE-I: BA response element-I
LRH-1: liver receptor homolog-1
FGF15: fibroblast growth factor 15
FGFR4: fibroblast growth factor receptor 4
TGR5: takeda G protein-coupled receptor 5
GLP-1: glucagon-like peptide-1
FGF19: fibroblast growth factor 19
LG: Ling-Gui-Zhu-Gan decoction
PCP: penthorum chinense pursh
PCBG: pinocembrin-7-O-β-D-glucoside
PCB: pinocembrin
HFD: high-fat diet
EGCG: epigallocatechin-3-gallate
BCRP: breast cancer resistance protein
PK: pharmacokinetic
FMT: fecal microbiota transplantation
LXRα: liver X receptor α
YTP: large-leaf yellow tea polysaccharide
SREBP-1c: sterol regulatory element-binding protein 1c
FASN: fatty acid synthase
ACC: acetyl-coA carboxylase
RCTs: randomized controlled trials
AS-IV: Astragaloside IV
7-KDCA: 7-keto-deoxycholic acid
SREBP1: sterol regulatory element-binding protein 1
Tα/β-MCA: tauro-α/β-muricholic acid
