# Supplementary material for: A critical review of natural products driven correction of bile acid dysregulation: a therapeutic strategy for nonalcoholic fatty liver disease
Source: Front Pharmacol. 2025 Nov 21;16:1640873. doi: 10.3389/fphar.2025.1640873 (PMC12678328; doi:10.3389/fphar.2025.1640873)
Supplement: Supplementary file 1 [file Table1.docx]

**Supplementary Table S1 Botanical composition and taxonomic validation of TCM formulas**

| **Number** | **TCM formula name** | **Chinese name** | **Botanical name (Authority)** | **Family** | **Ratio** | **Reference** |
| --- | --- | --- | --- | --- | --- | --- |
| 1 | Jiangzhi granule | Jiaogulan | *Gynostemma pentaphyllum* (Thunb.) *Makino* | Cucurbitaceae | 2.5 | Cao et al. (2022) |
|  |  | Huzhang | *Reynoutria japonica* Houtt. | Polygonaceae | 2.5 |  |
|  |  | Danshen | *Salvia miltiorrhiza* Bunge | Lamiaceae | 1.5 |  |
|  |  | Yinchen | *Artemisia capillaris* Thunb. | Asteraceae | 1.5 |  |
|  |  | Heye | *Nelumbo nucifera* Gaertn. | Nymphaeaceae | 1 |  |
| 2 | Ling-Gui-Zhu-Gan decoction  Ling-Gui-Zhu-Gan oral solution  Ling-Gui-Zhu-Gan decoction | Fuling | *Poria cocos* (Schw.) Wolf | Polyporaceae | 4 | Chen et al. (2024)  Wang et al. (2024a)  Dai et al. (2022) |
|  |  | Guizhi | *Cinnamomum cassia* (L.) J.Presl | Lauraceae | 3 |  |
|  |  | Baizhu | *Atractylodes macrocephala* Koidz. | Asteraceae | 3 |  |
|  |  | Gancao* | *Glycyrrhiza uralensis* Fisch. | Fabaceae | 2 |  |
| 3 | Zhuyu pill | Huanglian | *Coptis chinensis* Franch. | Ranunculaceae | 1 | Xu et al. (2023) |
|  |  | Wuzhuyu | *Tetradium ruticarpum* (A.Juss.) T.G.Hartley | Rutaceae | 1 |  |
| 4 |  | Ganhuangcao | *Penthorum chinense* Pursh | Saxifragaceae | - | Li et al. (2022a) |
| 5 | Huazhi-Rougan formula | Yinchen | *Artemisia capillaris* Thunb. | Asteraceae | - | Li et al. (2022c) |
|  |  | Juemingzi | *Senna obtusifolia* (L.) H.S.Irwin & Barneby | Fabaceae | - |  |
|  |  | Dahuang | *Rheum officinale* Baill. | Polygonaceae | - |  |
|  |  | Zexie | *Alisma plantago-aquatica subsp. orientale* (Sam.) Sam. | Alismataceae | - |  |
|  |  | Zhuling | *Polyporus umbellatus* (Pers.) Fr. | Polyporaceae | - |  |
|  |  | Shanzha | *Crataegus pinnatifida* Bunge | Rosaceae | - |  |
|  |  | Cangzhu | *Atractylodes lancea* (Thunb.) DC. | Asteraceae | - |  |
|  |  | Baizhu | *Atractylodes macrocephala* Koidz. | Asteraceae | - |  |
|  |  | Chenpi* | *Citrus reticulata* Blanco | Rutaceae | - |  |
|  |  | Gualou | *Trichosanthes kirilowii* Maxim. | Cucurbitaceae | - |  |
|  |  | Nvzhenzi | *Ligustrum lucidum* W.T.Aiton | Oleaceae | - |  |
|  |  | Mohanlian | *Eclipta prostrata* (L.) L. | Asteraceae | - |  |
|  |  | Gouqizi | *Lycium barbarum* L*.* | Solanaceae | - |  |
|  |  | Xiaoji | *Cirsium setosum* (Willd.) Besser | Asteraceae | - |  |
|  |  | Chaihu | *Bupleurum chinense* DC. | Apiaceae | - |  |
|  |  | Gancao* | *Glycyrrhiza uralensis* Fisch. | Fabaceae | - |  |
| 6 | Zexie-Baizhu Decoction | Zexie | *Alisma plantago-aquatica subsp. orientale* (Sam.) Sam. | Alismataceae | 5 | Shi et al. (2025) |
|  |  | Baizhu | *Atractylodes macrocephala* Koidz. | Asteraceae | 2 |  |
| 7 | Xiaohua Funing decoction | Zhuru | *Bambusa tuldoides* Munro | Poaceae | 2 | Li and Zhao. (2025) |
|  |  | Cangzhu | *Atractylodes lancea* (Thunb.) DC. | Asteraceae | 3 |  |
|  |  | Chaihu | *Bupleurum chinense* DC. | Apiaceae | 2 |  |
|  |  | Zhiqiao* | *Citrus aurantium* L. | Rutaceae | 3 |  |
|  |  | Huangqin | *Scutellaria baicalensis* Georgi | Lamiaceae | 2 |  |
|  |  | Jianghuang | *Curcuma longa* L*.* | Zingiberaceae | 3 |  |
|  |  | Yanhusuo | *Corydalis yanhusuo* (Y.H.Chou & Chun C.Hsu) W.T.Wang ex Z.Y.Su & C.Y.Wu | Papaveraceae | 3 |  |
|  |  | Baishao | *Paeonia lactiflora* Pall. | Paeoniaceae | 4 |  |
|  |  | Pugongying | *Taraxacum mongolicum* Hand.-Mazz. | Asteraceae | 4 |  |
|  |  | Shanzha | *Crataegus pinnatifida* Bunge | Rosaceae | 3 |  |
|  |  | Xiaomu | *Setaria italica* (L.) Beauv. | Poaceae | 4 |  |
|  |  | Cheqiancao | *Plantago asiatica* L. | Plantaginaceae | 3 |  |
| 8 | Gan-Jiang-Ling-Zhu Decoction | Gancao* | *Glycyrrhiza uralensis* Fisch. | Fabaceae | 1 | Ma et al. (2025) |
|  |  | Ganjiang | *Zingiber officinale* Roscoe | Zingiberaceae | 2 |  |
|  |  | Fuling | *Poria cocos* (Schw.) Wolf | Polyporaceae | 2 |  |
|  |  | Baizhu | *Atractylodes macrocephala* Koidz. | Asteraceae | 1 |  |
| 9 | Qiang-Gan formula  Qiang-Gan capsule | Yinchen | *Artemisia capillaris* Thunb. | Asteraceae | 10 | Li et al. (2020a)  Li et al. (2010) |
|  |  | Banlangen | *Isatis tinctoria* L. | Brassicaceae | 5 |  |
|  |  | Danggui | *Angelica sinensis* (Oliv.) Diels | Apiaceae | 5 |  |
|  |  | Baishao | *Paeonia lactiflora* Pall. | Paeoniaceae | 5 |  |
|  |  | Danshen | *Salvia miltiorrhiza* Bunge | Lamiaceae | 10 |  |
|  |  | Yujin | *Curcuma wenyujin* Y.H.Chen & C.Ling | Zingiberaceae | 5 |  |
|  |  | Huangqi | *Astragalus mongholicus* Bunge | Fabaceae | 10 |  |
|  |  | Dangshen | *Codonopsis pilosula* (Franch.) Nannf. | Campanulaceae | 5 |  |
|  |  | Zexie | *Alisma plantago-aquatica subsp. orientale* (Sam.) Sam. | Alismataceae | 5 |  |
|  |  | Huangjing | *Polygonatum kingianum* Collett & Hemsl. | Asparagaceae | 5 |  |
|  |  | Shendihuang | *Rehmannia glutinosa* (Gaertn.) DC. | Orobanchaceae | 5 |  |
|  |  | Shanyao | *Dioscorea polystachya* Turcz. | Dioscoreaceae | 5 |  |
|  |  | Shanzha | *Crataegus pinnatifida* Bunge | Rosaceae | 4 |  |
|  |  | Liushenqu* | *Persicaria hydropiper* (L.) Delarbre | Polygonaceae | 4 |  |
|  |  | Qinjiao | *Gentiana macrophylla* Pall. | Gentianaceae | 4 |  |
|  |  | Gancao* | *Glycyrrhiza uralensis* Fisch. | Fabaceae | 4 |  |
| 10 | Qinlian Hongqu Decoction | Huangqin | *Scutellaria baicalensis* Georgi | Lamiaceae | - | Zhang et al. (2024a) |
|  |  | Huanglian | *Coptis chinensis* Franch. | Ranunculaceae | - |  |
|  |  | Chenpi* | *Citrus reticulata* Blanco | Rutaceae | - |  |
|  |  | Hongqu | *Monascus purpureus* Went | Monascaceae | - |  |
|  |  | Zhigancao* | *Glycyrrhiza uralensis* Fisch. | Fabaceae | - |  |
|  |  | Baishao | *Paeonia lactiflora* Pall. | Paeoniaceae | - |  |
|  |  | Zhishi* | *Citrus aurantium* L. | Rutaceae | - |  |
|  |  | Heye | *Nelumbo nucifera* Gaertn. | Nymphaeaceae | - |  |
|  |  | Shengma | *Cimicifuga heracleifolia* Kom. | Ranunculaceae | - |  |
| 11 | Huaganjian decoction | Qingpi* | *Citrus reticulata* Blanco | Rutaceae | 4 | Do et al. (2025) |
|  |  | Chenpi* | *Citrus reticulata* Blanco | Rutaceae | 4 |  |
|  |  | Baishao | *Paeonia lactiflora* Pall. | Paeoniaceae | 4 |  |
|  |  | Mudanpi | *Paeonia suffruticosa* Andr. | Paeoniaceae | 3 |  |
|  |  | Zhizi | *Gardenia jasminoides* J. Ellis | Rubiaceae | 3 |  |
|  |  | Zexie | *Alisma plantago-aquatica subsp. orientale* (Sam.) Sam. | Alismataceae | 3 |  |
|  |  | Zhebeimu | *Fritillaria thunbergii* Miq. | Liliaceae | 5 |  |

Gancao* and Zhigancao*: The raw material for both Gancao and Zhigancao is the root and rhizome of *Glycyrrhiza uralensis* Fisch. They are distinguished by processing: Gancao is the raw, unprocessed form, whereas Zhigancao is prepared by stir-frying the raw herb with honey.

Liushenqu*: Liushenqu is a traditional Chinese medicine fermented preparation. In this table, its key fermented medicinal material, *Persicaria hydropiper (L.)* Delarbre, was selected as a representative for chemical composition.

Qingpi* and Chenpi*: The raw materials for both Qingpi and Chenpi are the dried pericarp of *Citrus reticulata* Blanco. They are distinguished by the fruit’s developmental stage at the time of collection: Chenpi is prepared from the peel of ripe fruits, whereas Qingpi is prepared from the peel of unripe, young fruits.

Zhiqiao* and Zhishi*: The raw materials for both Zhiqiao and Zhishi are the dried, unripe fruit of *Citrus aurantium* L. They are distinguished by the fruit's developmental stage at the time of collection: Zhishi is prepared from the smaller, younger fruit, whereas Zhiqiao is prepared from the larger, more developed fruit.
